# Correction to: “UDE DIATOMS in the Wild 2024”: a new image dataset of freshwater diatoms for training deep learning models

**DOI:** 10.1093/gigascience/giaf043

**Published:** 2025-03-26

This is a correction to: Aishwarya Venkataramanan, Michael Kloster, Andrea Burfeid-Castellanos, Mimoza Dani, Ntambwe A S Mayombo, Danijela Vidakovic, Daniel Langenkämper, Mingkun Tan, Cedric Pradalier, Tim Nattkemper, Martin Laviale, Bánk Beszteri, “UDE DIATOMS in the Wild 2024”: a new image dataset of freshwater diatoms for training deep learning models, *GigaScience*, Volume 13, 2024, 10.1093/gigascience/giae087

In the originally published version of the manuscript the authors mistakenly referred to deep learning experiment 2 as "semi-supervised learning" instead of "self-supervised learning". Emendation has been made to read: "self-supervised" throughout the paper.

