# Supplementary material for: “UDE DIATOMS in the Wild 2024”: a new image dataset of freshwater diatoms for training deep learning models
Source: Gigascience. 2024 Nov 28;13:giae087. doi: 10.1093/gigascience/giae087 (PMC11604061; doi:10.1093/gigascience/giae087)
Supplement: giae087_Supplementary_Files [file giae087_supplementary_files.zip › Supplement Table 2 - Species abundance.docx]

# Supplement Table 2: Species abundance

|  | **Species** | **Abundance** |
| --- | --- | --- |
| 1 | *Achnanthidium minutissimum* | 15,573 |
| 2 | *Discostella pseudostelligera* | 3,527 |
| 3 | *Amphora pediculus* | 3,099 |
| 4 | *Achnanthidium jackii* | 2,875 |
| 5 | *Navicula gregaria* | 2,324 |
| 6 | *Navicula lanceolata* | 2,274 |
| 7 | *Nitzschia soratensis* | 2,055 |
| 8 | *Planothidium lanceolatum* | 1,942 |
| 9 | *Nitzschia dissipata* | 1,886 |
| 10 | *Cocconeis placentula* | 1,822 |
| 11 | *Achnanthidium pyrenaicum* | 1,569 |
| 12 | *Planothidium frequentissimum* | 1,369 |
| 13 | *Nitzschia inconspicua* | 1,314 |
| 14 | *Sellaphora nigri* | 951 |
| 15 | *Cyclotella meduanae* | 941 |
| 16 | *Nitzschia palea* | 937 |
| 17 | *Cyclotella meneghiniana (Stephanocyclus meneghiniana)* | 892 |
| 18 | *Stephanodiscus lacustris* | 808 |
| 19 | *Achnanthidium atomoides* | 798 |
| 20 | *Gomphonella olivacea* | 781 |
| 21 | *Surirella brebissonii* | 663 |
| 22 | *Rhoicosphenia abbreviata* | 617 |
| 23 | *Navicula veneta* | 599 |
| 24 | *Mayamaea permitis* | 592 |
| 25 | *Denticula tenuis* | 589 |
| 26 | *Navicula germainii* | 531 |
| 27 | *Luticola frequentissima* | 493 |
| 28 | *Gyrosigma acuminatum* | 468 |
| 29 | *Gomphonema parvulum* | 451 |
| 30 | *Navicula cryptotenella* | 446 |
| 31 | *Crenotia rumrichorum* | 417 |
| 32 | *Humidophila contemnata* | 410 |
| 33 | *Meridion circulare* | 409 |
| 34 | *Gomphonema micropus* | 407 |
| 35 | *Navicula recens* | 361 |
| 36 | *Aulacoseira granulata* | 352 |
| 37 | *Diatoma tenuis* | 347 |
| 38 | *Fragilaria famelica* | 339 |
| 39 | *Amphora indistincta* | 330 |
| 40 | *Cyclotella atomus* | 327 |
| 41 | *Fragilaria deformis* | 324 |
| 42 | *Seminavis strigosa* | 324 |
| 43 | *Melosira varians* | 301 |
| 44 | *Achnanthidium saprophilum* | 287 |
| 45 | *Achnanthidium lineare* | 280 |
| 46 | *Navicula tripunctata* | 262 |
| 47 | *Achnanthidium rivulare* | 261 |
| 48 | *Fragilaria rinoi* | 255 |
| 49 | *Fistulifera saprophila* | 247 |
| 50 | *Nitzschia frustulum* | 240 |
| 51 | *Cyclostephanos invisitatus* | 234 |
| 52 | *Humidophila aerophila* | 234 |
| 53 | *Navicula rostellata* | 225 |
| 54 | *Surirella minuta* | 216 |
| 55 | *Navicula cryptocephala* | 214 |
| 56 | *Gomphonema sp. 1* | 211 |
| 57 | *Navicula antonii* | 209 |
| 58 | *Encyonema minutum* | 207 |
| 59 | *Amphora inariensis* | 201 |
| 60 | *Fragilaria vaucheriae* | 198 |
| 61 | *Sellaphora crassulexigua* | 197 |
| 62 | *Gomphonema pumilum* | 192 |
| 63 | *Nitzschia adamata* | 191 |
| 64 | *Nitzschia sociabilis* | 191 |
| 65 | *Navicula metareichardtiana = reichardtiana* | 190 |
| 66 | *Humidophila simplex* | 188 |
| 67 | *Nitzschia fonticola* | 169 |
| 68 | *Sellaphora pupula* | 169 |
| 69 | *Conticribra weissflogii* | 165 |
| 70 | *Stephanodiscus parvus* | 165 |
| 71 | *Nitzschia supralitorea* | 162 |
| 72 | *Encyonema silesiacum* | 161 |
| 73 | *Cocconeis pediculus* | 156 |
| 74 | *Reimeria uniseriata* | 155 |
| 75 | *Achnanthidium straubianum* | 153 |
| 76 | *Ulnaria ulna* | 151 |
| 77 | *Achnanthidium subatomus* | 148 |
| 78 | *Gomphonema calcifugum* | 144 |
| 79 | *Humidophila contenta* | 135 |
| 80 | *Achnanthidium eutrophilum* | 133 |
| 81 | *Cyclostephanos dubius* | 132 |
| 82 | *Navicula upsaliensis* | 132 |
| 83 | *Nitzschia vermicularis* | 132 |
| 84 | *Sellaphora raederae* | 132 |
| 85 | *Caloneis lancettula* | 126 |
| 86 | *Tabularia fasciculata* | 124 |
| 87 | *Pantocksekiella ocellata* | 123 |
| 88 | *Encyonema ventricosum* | 119 |
| 89 | *Gomphonema minutum* | 119 |
| 90 | *Reimeria sinuata* | 118 |
| 91 | *Gomphonema elegantissimum* | 115 |
| 92 | *Sellaphora seminulum* | 114 |
| 93 | *Navicula cryptotenelloides* | 113 |
| 94 | *Navicula slesvicensis* | 113 |
| 95 | *Achnanthidium delmontii* | 112 |
| 96 | *Navicula erifuga* | 110 |
| 97 | *Stephanodiscus hantzschii* | 109 |
| 98 | *Diatoma moniliformis* | 107 |
| 99 | *Navicula simulata* | 107 |
| 100 | *Navicymbula pusilla* | 105 |
| 101 | *Encyonopsis minuta* | 102 |
| 102 | *Sellaphora saugerresii* | 97 |
| 103 | *Nitzschia recta* | 96 |
| 104 | *Navicula amphiceropsis* | 94 |
| 105 | *Gomphonema utae* | 93 |
| 106 | *Diatoma vulgaris* | 90 |
| 107 | *Nitzschia paleacea* | 85 |
| 108 | *Planothidium caputium* | 84 |
| 109 | *Navicula caterva* | 81 |
| 110 | *Achnanthidium kranzii* | 80 |
| 111 | *Nitzschia acicularis* | 80 |
| 112 | *Nitzschia amphibia* | 80 |
| 113 | *Achnanthidium microcephalum* | 79 |
| 114 | *Nitzschia pusilla* | 79 |
| 115 | *Ctenophora pulchella* | 76 |
| 116 | *Nitzschia archibaldii* | 76 |
| 117 | *Nitzschia intermedia* | 75 |
| 118 | *Platessa oblongella* | 73 |
| 119 | *Bacillaria paxillifera* | 71 |
| 120 | *Craticula subminuscula* | 71 |
| 121 | *Halamphora montana* | 71 |
| 122 | *Gomphonema minusculum* | 70 |
| 123 | *Luticola mutica* | 70 |
| 124 | *Rhopalodia operculata* | 70 |
| 125 | *Rhoicosphenia lacustris* | 67 |
| 126 | *Nitzschia agnita* | 65 |
| 127 | *Cyclotella distinguenda* | 62 |
| 128 | *Lemnicola hungarica* | 62 |
| 129 | *Aulacoseira ambigua* | 61 |
| 130 | *Entomoneis paludosa* | 58 |
| 131 | *Asterionella formosa* | 57 |
| 132 | *Fragilaria capucina* | 57 |
| 133 | *Gomphonema tergestinum* | 57 |
| 134 | *Nitzschia tubicola* | 56 |
| 135 | *Ulnaria acus* | 56 |
| 136 | *Fragilaria recapitellata* | 55 |
| 137 | *Nitzschia microcephala* | 54 |
| 138 | *Hippodonta capitata* | 53 |
| 139 | *Nitzschia frustulum var. subsalina* | 53 |
| 140 | *Planothidium hauckianum* | 53 |
| 141 | *Psammothidium grischunum* | 53 |
| 142 | *Luticola micra* | 52 |
| 143 | *Luticola rotunda* | 51 |
| 144 | *Navicula capitatoradiata* | 50 |
| 145 | *Humidophila brekkaensis* | 49 |
| 146 | *Thalassiosira pseudonana* | 49 |
| 147 | *Achnanthidium exile* | 48 |
| 148 | *Fragilaria rumpens* | 47 |
| 149 | *Nitzschia linearis* | 46 |
| 150 | *Odontidium mesodon* | 46 |
| 151 | *Halamphora veneta* | 45 |
| 152 | *Staurosirella pinnata* | 45 |
| 153 | *Navicula vandamii* | 44 |
| 154 | *Nitzschia perminuta* | 44 |
| 155 | *Discostella asterocostata* | 43 |
| 156 | *Gomphonema lateripunctatum* | 43 |
| 157 | *Parlibellus protractoides* | 43 |
| 158 | *Fragilaria gracilis* | 42 |
| 159 | *Navicula trivialis* | 42 |
| 160 | *Nitzschia austriaca* | 42 |
| 161 | *Halamphora coffeaeformis* | 41 |
| 162 | *Amphora copulata* | 39 |
| 163 | *Craticula halophila* | 39 |
| 164 | *Craticula molestiformis* | 39 |
| 165 | *Frustulia creuzburgensis* | 37 |
| 166 | *Mayamaea atomus* | 36 |
| 167 | *Psammothidium subatomoides* | 36 |
| 168 | *Navicula supergregaria* | 35 |
| 169 | *Surirella angusta* | 35 |
| 170 | *Aulacoseira pusilla* | 34 |
| 171 | *Staurosira venter* | 34 |
| 172 | *Gomphonema graciledictum* | 33 |
| 173 | *Gomphonema parvuliforme* | 33 |
| 174 | *Gomphonema parvulius* | 33 |
| 175 | *Humidophila gallica* | 33 |
| 176 | *Mayamaea fossalis* | 33 |
| 177 | *Adlafia bryophila* | 32 |
| 178 | *Amphora ovalis* | 31 |
| 179 | *Cymbella affinis* | 31 |
| 180 | *Psammothidium lauenburgianum* | 31 |
| 181 | *Nitzschia hantzschiana* | 29 |
| 182 | *Placoneis symmetrica* | 29 |
| 183 | *Gyrosigma sciotense* | 28 |
| 184 | *Nitzschia aurariae* | 28 |
| 185 | *Parlibellus protracta = protractus* | 28 |
| 186 | *Achnanthidium affine* | 27 |
| 187 | *Humidophila perpusilla* | 27 |
| 188 | *Platessa conspicua* | 27 |
| 189 | *Fragilaria pectinalis* | 26 |
| 190 | *Fragilaria tenera* | 26 |
| 191 | *Cymbella tumida* | 25 |
| 192 | *Encyonopsis subminuta* | 25 |
| 193 | *Fragilaria microvaucheriae* | 25 |
| 194 | *Frustulia vulgaris* | 25 |
| 195 | *Navicula praeterita* | 25 |
| 196 | *Tryblionella hungarica* | 25 |
| 197 | *Achnanthidium latecephalum* | 24 |
| 198 | *Cymbella excisiformis* | 24 |
| 199 | *Fistulifera pelliculosa* | 24 |
| 200 | *Nitzschia denticula* | 24 |
| 201 | *Pseudostaurosira brevistriata* | 24 |
| 202 | *Tryblionella apiculata* | 24 |
| 203 | *Adlafia minuscula* | 23 |
| 204 | *Cymbella excisa* | 23 |
| 205 | *Diatoma ehrenbergii* | 23 |
| 206 | *Grunowia solgensis* | 23 |
| 207 | *Fallacia subhamulata* | 22 |
| 208 | *Gomphonema subclavatum* | 22 |
| 209 | *Pseudostaurosira parasitica* | 22 |
| 210 | *Nitzschia fossilis* | 21 |
| 211 | *Nitzschia solgensis* | 21 |
| 212 | *Planothidium delicatulum* | 21 |
| 213 | *Cymatopleura solea = Surirella librile* | 20 |
| 214 | *Epithemia sorex* | 20 |
| 215 | *Navicula lacuum* | 20 |
| 216 | *Navicula salinarum* | 20 |
| 217 | *Navicula tenelloides* | 20 |
| 218 | *Stephanodiscus minutulus* | 20 |
| 219 | *Caloneis vasileyevae* | 19 |
| 220 | *Discostella stelligera* | 19 |
| 221 | *Fragilaria pararumpens* | 19 |
| 222 | *Hannaea arcus* | 19 |
| 223 | *Skeletonema potamos* | 19 |
| 224 | *Diatoma problematica* | 18 |
| 225 | *Diploneis separanda* | 18 |
| 226 | *Gomphonema campoduense* | 18 |
| 227 | *Nitzschia clausii* | 18 |
| 228 | *Nitzschia tenuis* | 18 |
| 229 | *Stauroneis kriegerii* | 18 |
| 230 | *Achnanthidium druartii* | 17 |
| 231 | *Eolimna tantula* | 17 |
| 232 | *Nitzschia acidoclinata* | 17 |
| 233 | *Tryblionella levidensis* | 17 |
| 234 | *Diploneis praetermissa* | 16 |
| 235 | *Gomphonema innocens* | 16 |
| 236 | *Navicula viridulacalcis* | 16 |
| 237 | *Tryblionella calida* | 16 |
| 238 | *Astartiella bahusiensis* | 15 |
| 239 | *Gomphonema angustatum* | 15 |
| 240 | *Nitzschia capitellata* | 15 |
| 241 | *Fallacia pygmaea* | 14 |
| 242 | *Navicula radiosa* | 14 |
| 243 | *Encyonema caespitosum* | 13 |
| 244 | *Sellaphora stroemii* | 13 |
| 245 | *Stauroneis smithii* | 13 |
| 246 | *Achnanthidium catenatum* | 12 |
| 247 | *Berkeleya rutilans* | 12 |
| 248 | *Craticula simplex* | 12 |
| 249 | *Gomphonema angustivalva* | 12 |
| 250 | *Luticola muticoides* | 12 |
| 251 | *Navicula cincta* | 12 |
| 252 | *Psammothidium helveticum* | 12 |
| 253 | *Sellaphora utermoehlii* | 12 |
| 254 | *Diploneis oblongellopsis* | 11 |
| 255 | *Sellaphora verecundiae* | 11 |
| 256 | *Achnanthidium gracillimum* | 10 |
| 257 | *Achnanthidium rosenstockii* | 10 |
| 258 | *Achnanthidium sieminskae* | 10 |
| 259 | *Cymbopleura amphicephala* | 10 |
| 260 | *Encyonopsis krammeri* | 10 |
| 261 | *Encyonopsis microcephala* | 10 |
| 262 | *Navicula cari* | 10 |
| 263 | *Nitzschia heufleriana* | 10 |
| 264 | *Pseudofallacia tenera* | 10 |
| 265 | *Achnanthidium caledonicum* | 9 |
| 266 | *Actinocyclus normanii* | 9 |
| 267 | *Cyclotella costei* | 9 |
| 268 | *Diploneis oculata* | 9 |
| 269 | *Gomphonema pseudobohemicum* | 9 |
| 270 | *Gomphonema sphenovertex* | 9 |
| 271 | *Gyrosigma macrum* | 9 |
| 272 | *Karayevia laterostrata* | 9 |
| 273 | *Luticola sp. 2* | 9 |
| 274 | *Prestauroneis integra* | 9 |
| 275 | *Rossithidium petersenii* | 9 |
| 276 | *Tryblionella salinarum* | 9 |
| 277 | *Achnanthidium exiguum (Gogorevia exilis)* | 8 |
| 278 | *Craticula accomoda* | 8 |
| 279 | *Craticula buderi* | 8 |
| 280 | *Diploneis gallica* | 8 |
| 281 | *Gomphonema cymbelliclinum* | 8 |
| 282 | *Halamphora normanii* | 8 |
| 283 | *Luticola sp. 1* | 8 |
| 284 | *Nitzschia vermicularoides* | 8 |
| 285 | *Psammothidium microscopicum* | 8 |
| 286 | *Pseudofallacia monoculata* | 8 |
| 287 | *Surirella suecica* | 8 |
| 288 | *Tryblionella angustatula* | 8 |
| 289 | *Caloneis fontinalis* | 7 |
| 290 | *Gomphonema angustum* | 7 |
| 291 | *Gomphonema exilissimum* | 7 |
| 292 | *Gomphonema extentum* | 7 |
| 293 | *Gomphonema hristovskii* | 7 |
| 294 | *Surirella tenera* | 7 |
| 295 | *Achnanthidium pfisteri* | 6 |
| 296 | *Cymbella parva* | 6 |
| 297 | *Eunotia exigua* | 6 |
| 298 | *Gomphonema capitatum* | 6 |
| 299 | *Gomphonema clavatum* | 6 |
| 300 | *Hantzschia amphioxys* | 6 |
| 301 | *Karayevia clevei* | 6 |
| 302 | *Kolbesia ploenensis* | 6 |
| 303 | *Luticola goeppertiana* | 6 |
| 304 | *Luticola muticopsis* | 6 |
| 305 | *Luticola saprophila* | 6 |
| 306 | *Navicula subalpina* | 6 |
| 307 | *Nitzschia acula* | 6 |
| 308 | *Nitzschia sigma* | 6 |
| 309 | *Pinnularia subrupestris* | 6 |
| 310 | *Placoneis undulata* | 6 |
| 311 | *Planothidium minutissimum* | 6 |
| 312 | *Psammothidium daoense* | 6 |
| 313 | *Rhopalodia gibba* | 6 |
| 314 | *Tryblionella debilis* | 6 |
| 315 | *Cylindrotheca gracilis* | 5 |
| 316 | *Cymbella compacta* | 5 |
| 317 | *Diadesmis confervacea* | 5 |
| 318 | *Fragilariforma virescens* | 5 |
| 319 | *Gomphonema dojranense* | 5 |
| 320 | *Gomphonema productum* | 5 |
| 321 | *Gomphosphenia fontinalis* | 5 |
| 322 | *Gyrosigma obtusatum* | 5 |
| 323 | *Hippodonta hungarica* | 5 |
| 324 | *Hippodonta neglecta* | 5 |
| 325 | *Navicula kotschyi* | 5 |
| 326 | *Navicula notha* | 5 |
| 327 | *Navicula oblonga* | 5 |
| 328 | *Nitzschia communis* | 5 |
| 329 | *Nitzschia paleaeformis* | 5 |
| 330 | *Nitzschia thermaloides* | 5 |
| 331 | *Placoneis anglica* | 5 |
| 332 | *Simonsenia delognei* | 5 |
| 333 | *Stauroneis thermicola* | 5 |
| 334 | *Staurosirella oldenburgiana* | 5 |
| 335 | *Caloneis aerophila* | 4 |
| 336 | *Caloneis amphisbaena* | 4 |
| 337 | *Caloneis silicula* | 4 |
| 338 | *Chamaepinnularia muscicola* | 4 |
| 339 | *Craticula minusculoides* | 4 |
| 340 | *Cymbella kolbei* | 4 |
| 341 | *Cymbopleura frequens* | 4 |
| 342 | *Diploneis marginastriata* | 4 |
| 343 | *Eunotia bilunaris* | 4 |
| 344 | *Fallacia insociabilis* | 4 |
| 345 | *Fragilaria neointermedia* | 4 |
| 346 | *Fragilariforma bicapitata* | 4 |
| 347 | *Gomphonema occultum* | 4 |
| 348 | *Luticola ventricosa* | 4 |
| 349 | *Navicula rhynchotella* | 4 |
| 350 | *Navicula salinicola* | 4 |
| 351 | *Navicula vilaplanii* | 4 |
| 352 | *Nitzschia dealpina* | 4 |
| 353 | *Nitzschia dubia* | 4 |
| 354 | *Nitzschia filiformis* | 4 |
| 355 | *Nitzschia gracilis* | 4 |
| 356 | *Nitzschia lacuum* | 4 |
| 357 | *Nitzschia sigmoidea* | 4 |
| 358 | *Nitzschia solita* | 4 |
| 359 | *Sellaphora bacillum* | 4 |
| 360 | *Surirella linearis* | 4 |
| 361 | *Surirella terricola* | 4 |
| 362 | *Tryblionella angustata* | 4 |
| 363 | *Ulnaria grunowii* | 4 |
| 364 | *Amphipleura pellucida* | 3 |
| 365 | *Amphora aequalis* | 3 |
| 366 | *Brachysira neoexilis* | 3 |
| 367 | *Chamaepinnularia submuscicola* | 3 |
| 368 | *Crenotia thermalis* | 3 |
| 369 | *Cymbellonitzschia diluviana* | 3 |
| 370 | *Diploneis pseudopetersenii* | 3 |
| 371 | *Fallacia lenzii* | 3 |
| 372 | *Fragilaria austriaca* | 3 |
| 373 | *Fragilaria parasitica* | 3 |
| 374 | *Fragilaria radians* | 3 |
| 375 | *Fragilaria saxoplanctonica* | 3 |
| 376 | *Gomphonema clavatulum* | 3 |
| 377 | *Gomphosphenia holmquistii* | 3 |
| 378 | *Humidophila vidalii* | 3 |
| 379 | *Luticola imbricata* | 3 |
| 380 | *Navicula associata* | 3 |
| 381 | *Navicula densilineolata* | 3 |
| 382 | *Navicula oligotraphenta* | 3 |
| 383 | *Nitzschia alpina* | 3 |
| 384 | *Nitzschia bulnheimiana* | 3 |
| 385 | *Nitzschia valdestriata* | 3 |
| 386 | *Nitzschia wuellerstorfii* | 3 |
| 387 | *Sellaphora absoluta* | 3 |
| 388 | *Staurosira binodis* | 3 |
| 389 | *Surirella ovalis* | 3 |
| 390 | *Tryblionella brunoi* | 3 |
| 391 | *Achnanthes coarctata* | 2 |
| 392 | *Achnanthidium neomicrocephalum* | 2 |
| 393 | *Achnanthidium trinode* | 2 |
| 394 | *Amphora lange-bertalotii* | 2 |
| 395 | *Caloneis macedonica* | 2 |
| 396 | *Craticula ambigua* | 2 |
| 397 | *Cymbella vulgata* | 2 |
| 398 | *Diploneis carloswetzelii* | 2 |
| 399 | *Diploneis fontanella* | 2 |
| 400 | *Diploneis metapumila* | 2 |
| 401 | *Diploneis puellafallax* | 2 |
| 402 | *Encyonema lange-bertalotii* | 2 |
| 403 | *Encyonema vulgare* | 2 |
| 404 | *Eunotia valida* | 2 |
| 405 | *Fragilaria mesolepta* | 2 |
| 406 | *Geissleria acceptata* | 2 |
| 407 | *Gomphonella olivaceoides var. desestriata* | 2 |
| 408 | *Gomphonema acuminatum* | 2 |
| 409 | *Gomphonema amoenum* | 2 |
| 410 | *Gomphonema jablanicense* | 2 |
| 411 | *Gyrosigma kuetzingii* | 2 |
| 412 | *Hippodonta pumila* | 2 |
| 413 | *Lindavia radiosa* | 2 |
| 414 | *Luticola acidoclinata* | 2 |
| 415 | *Luticola nivalis* | 2 |
| 416 | *Luticola similis* | 2 |
| 417 | *Navicula hofmanniae* | 2 |
| 418 | *Navicula pargemina* | 2 |
| 419 | *Navicula perminuta* | 2 |
| 420 | *Navicula rhynchocephala* | 2 |
| 421 | *Navicula viridula* | 2 |
| 422 | *Neidiomorpha binodiformis* | 2 |
| 423 | *Neidium productum* | 2 |
| 424 | *Nitzschia diversa* | 2 |
| 425 | *Nitzschia frequens* | 2 |
| 426 | *Nitzschia reversa* | 2 |
| 427 | *Pinnularia schoenfelderi* | 2 |
| 428 | *Placoneis pseudanglica* | 2 |
| 429 | *Planothidium amphibium* | 2 |
| 430 | *Planothidium cryptolanceolatum* | 2 |
| 431 | *Planothidium rostratoholarcticum* | 2 |
| 432 | *Pleurosira laevis* | 2 |
| 433 | *Psammothidium bioretii* | 2 |
| 434 | *Punctastriata lancettula* | 2 |
| 435 | *Sellaphora rotunda* | 2 |
| 436 | *Sellaphora saprotolerans* | 2 |
| 437 | *Stauroneis separanda* | 2 |
| 438 | *Staurosira construens* | 2 |
| 439 | *Staurosira subsalina* | 2 |
| 440 | *Stephanocyclus meneghiniana* | 2 |
| 441 | *Achnanthidium atomus* | 1 |
| 442 | *Achnanthidium temniskovae* | 1 |
| 443 | *Amphora hemicyclus* | 1 |
| 444 | *Amphora minutissima* | 1 |
| 445 | *Caloneis bacillum* | 1 |
| 446 | *Caloneis schumanniana* | 1 |
| 447 | *Caloneis tenuis* | 1 |
| 448 | *Cocconeis neothumensis* | 1 |
| 449 | *Cyclotella ocellata* | 1 |
| 450 | *Cylindrotheca closterium* | 1 |
| 451 | *Cymbella convexa* | 1 |
| 452 | *Cymbella diminuta = Cymbopleura diminuta* | 1 |
| 453 | *Cymbella lange-bertalotii* | 1 |
| 454 | *Cymbella omaniana* | 1 |
| 455 | *Cymbella superparva* | 1 |
| 456 | *Cymbopleura rhomboidea* | 1 |
| 457 | *Denticula subtilis* | 1 |
| 458 | *Diatoma polonica* | 1 |
| 459 | *Didymosphenia geminata* | 1 |
| 460 | *Diploneis krammeri* | 1 |
| 461 | *Diploneis modica* | 1 |
| 462 | *diploneis navahoarum* | 1 |
| 463 | *Discostella woltereckii* | 1 |
| 464 | *Encyonema hebridicum* | 1 |
| 465 | *Encyonema leibleinii* | 1 |
| 466 | *Encyonema prostratum* | 1 |
| 467 | *Eunotia nymanniana* | 1 |
| 468 | *Eunotia paludosa* | 1 |
| 469 | *Eunotia paratridentula* | 1 |
| 470 | *Eunotia tenella* | 1 |
| 471 | *Fallacia lucinensis* | 1 |
| 472 | *Fragilariforma atomus* | 1 |
| 473 | *Geissleria decussis* | 1 |
| 474 | *Gomphonema auritum* | 1 |
| 475 | *Gomphonema hebridense* | 1 |
| 476 | *Gomphonema jadwigiae* | 1 |
| 477 | *Gomphonema pala* | 1 |
| 478 | *Gomphonema subangustatum* | 1 |
| 479 | *Gomphonema subtergestinum* | 1 |
| 480 | *Gomphonema varioreduncum* | 1 |
| 481 | *Gomphonema variscohercynicum* | 1 |
| 482 | *Grunowia tabellaria* | 1 |
| 483 | *Halamphora oligotraphenta* | 1 |
| 484 | *Hippodonta linearis* | 1 |
| 485 | *Humidophila paracontenta* | 1 |
| 486 | *Luticola andina* | 1 |
| 487 | *Luticola imbricatiformis* | 1 |
| 488 | *Luticola lanceolata* | 1 |
| 489 | *Luticola nana* | 1 |
| 490 | *Luticola pseudoimbrincata* | 1 |
| 491 | *Luticola ventriconfusa* | 1 |
| 492 | *Navicula cryptocephaloides* | 1 |
| 493 | *Navicula escambia* | 1 |
| 494 | *Navicula leptostriata* | 1 |
| 495 | *Navicula libonensis* | 1 |
| 496 | *Navicula oppugnata* | 1 |
| 497 | *Navicula sp. 1* | 1 |
| 498 | *Navicula sparsistriata* | 1 |
| 499 | *Neidium affine* | 1 |
| 500 | *Nitzschia fibulafissa* | 1 |
| 501 | *Nitzschia graciliformis* | 1 |
| 502 | *Nitzschia oligotraphenta* | 1 |
| 503 | *Nitzschia pura* | 1 |
| 504 | *Nitzschia regula* | 1 |
| 505 | *Nitzschia sinuata* | 1 |
| 506 | *Nitzschia subtilis* | 1 |
| 507 | *Nitzschia umbonata* | 1 |
| 508 | *Nupela lapidosa* | 1 |
| 509 | *Pantocksekiella comensis* | 1 |
| 510 | *Pantocksekiella delicatula* | 1 |
| 511 | *Paraplaconeis minor* | 1 |
| 512 | *Pinnularia appendiculata* | 1 |
| 513 | *Pinnularia lundii* | 1 |
| 514 | *Pinnularia microstauron* | 1 |
| 515 | *Pinnularia obscura* | 1 |
| 516 | *Pinnularia sinistra* | 1 |
| 517 | *Pinnularia viridiformis* | 1 |
| 518 | *Placoneis clementispronina* | 1 |
| 519 | *Placoneis gastrum* | 1 |
| 520 | *Placoneis paraelginensis* | 1 |
| 521 | *Planothidium cavilanceolatum* | 1 |
| 522 | *Planothidium dubium* | 1 |
| 523 | *Planothidium granum* | 1 |
| 524 | *Platessa holsatica* | 1 |
| 525 | *Psammothidium punctulatum* | 1 |
| 526 | *Pseudostaurosira trinorii* | 1 |
| 527 | *Puncticulata radiosa* | 1 |
| 528 | *Sellaphora mutatoides* | 1 |
| 529 | *Sellaphora parapupula* | 1 |
| 530 | *Sellaphora pseudopupula* | 1 |
| 531 | *Sellaphora schadei* | 1 |
| 532 | *Stauroforma exiguiformis* | 1 |
| 533 | *Stauroneis leguminopsis* | 1 |
| 534 | *Stauroneis parathermicola* | 1 |
| 535 | *Staurosirella lapponica* | 1 |
| 536 | *Surirella angustata* | 1 |
| 537 | *Surirella biseriata* | 1 |
| 538 | *Surirella splendida* | 1 |
| 539 | *Tabellaria flocculosa* | 1 |
| 540 | *Tabularia tabulata* | 1 |
| 541 | *Tetracyclus rupestris* | 1 |
| 542 | *Tryblionella littoralis* | 1 |
