# Supplementary material for: “UDE DIATOMS in the Wild 2024”: a new image dataset of freshwater diatoms for training deep learning models
Source: Gigascience. 2024 Nov 28;13:giae087. doi: 10.1093/gigascience/giae087 (PMC11604061; doi:10.1093/gigascience/giae087)
Supplement: giae087_GIGA-D-24-00056_Revision_2 [file giae087_giga-d-24-00056_revision_2.pdf]

GigaScience

"UDE DIATOMS in the Wild 2024": A new image dataset of freshwater diatoms for training deep learning models

--Manuscript Draft--

**Manuscript Number:**	GIGA-D-24-00056R2
**Full Title:**	"UDE DIATOMS in the Wild 2024": A new image dataset of freshwater diatoms for training deep learning models
**Article Type:**	Data Note
**Funding Information:**	Deutsche Forschungsgemeinschaft (463395318) — Dr. Michael Kloster, Dr. Daniel Langenkämper, Prof. Dr. Tim Nattkemper, Prof. Dr. Bank Beszteri<br>Deutsche Forschungsgemeinschaft (426547801) — MSc Ntambwe Albert Serge Mayombo, Prof. Dr. Bank Beszteri<br>Horizon 2020 (201980E121) — Dr. Andrea Burfeid-Castellanos<br>Alexander von Humboldt-Stiftung (SRB 1221045 HFST-P) — Dr. Danijela Vidakovic<br>Agence Nationale de la Recherche (ANR-20-THIA-0010) — Dr. Aishwarya Venkataramanan<br>Région Grand-Est, France — Dr. Aishwarya Venkataramanan<br>Horizon 2020 (101058625) — Dr. Aishwarya Venkataramanan, Prof. Dr. Martin Laviale
**Abstract:**	Background: Diatoms are microalgae with finely ornamented microscopic silica shells. Their taxonomic identification by light microscopy is routinely used as part of community ecological research as well as ecological status assessment of aquatic ecosystems, and a need for digitalisation of these methods has long been recognized. Alongside their high taxonomic and morphological diversity, several other factors make diatoms highly challenging for deep learning-based identification using light microscopy images. These include a) an unusually high intra-class variability combined with small between-class differences; b) a rather different visual appearance of specimens depending on their orientation on the microscope slide; and c) the limited availability of diatom experts for accurate taxonomic annotation.<br>Findings: We present the largest diatom image dataset thus far, aimed at facilitating the application and benchmarking of innovative deep learning methods to the diatom identification problem on realistic research data, "UDE DIATOMS in the Wild 2024". The dataset contains 83,570 images of 611 diatom taxa, 101 of which are represented by at least 100 examples, and 144 by at least 50 examples each. We showcase this dataset in two innovative analyses that address individual aspects of the above challenges using subclustering to deal with visually heterogeneous classes, out-of-distribution sample detection and semi-supervised learning.<br>Conclusions: The problem of image-based identification of diatoms is both important for environmental research, and challenging from the machine learning perspective. By making available the so far largest image data set, accompanied by innovative analyses, this contribution will facilitate addressing these points by the scientific community.
**Corresponding Author:**	Bank Beszteri<br>University of Duisburg-Essen Faculty of Biology: Universitat Duisburg-Essen Fakultat fur Biologie<br>Essen, GERMANY
**Corresponding Author Secondary Information:**	
**Corresponding Author's Institution:**	University of Duisburg-Essen Faculty of Biology: Universitat Duisburg-Essen Fakultat fur Biologie

Powered by Editorial Manager® and ProduXion Manager® from Aries Systems Corporation

**Corresponding Author's Secondary Institution:**	
**First Author:**	Aishwarya Venkataramanan
**First Author Secondary Information:**	
**Order of Authors:**	Aishwarya Venkataramanan
	Michael Kloster
	Andrea Burfeid-Castellanos
	Mimoza Dani
	Ntambwe Albert Serge Mayombo
	Danijela Vidakovic
	Daniel Langenkämper
	Mingkun Tan
	Cedric Pradalier
	Tim Nattkemper
	Martin Laviale
	Bank Beszteri
**Order of Authors Secondary Information:**	
**Response to Reviewers:**	Dear Hans,<br><br>I uploaded the manuscript file after implementing the requested changes.<br>As to your question about the redundance between the Zenodo and Kaggle repositories: the co-authors would like to have the data on Kaggle as a "living" version of the data set that can be updated in the future. That is the reason for not freezing it with a DOI (I previously dicussed this point with the database editor as well). So I kept both the Zenodo DOI, as well as the Kaggle URL. I hope this is ok also from your side.<br><br>Best regards,<br>Bank
**Additional Information:**	
**Question**	**Response**
Are you submitting this manuscript to a special series or article collection?	No
**Experimental design and statistics**<br><br>Full details of the experimental design and statistical methods used should be given in the Methods section, as detailed in our Minimum Standards Reporting Checklist. Information essential to interpreting the data presented should be made available in the figure legends.<br><br>Have you included all the information requested in your manuscript?	No

Powered by Editorial Manager® and ProduXion Manager® from Aries Systems Corporation

If not, please give reasons for any omissions below.<br><br>as follow-up to "**Experimental design and statistics**<br><br>Full details of the experimental design and statistical methods used should be given in the Methods section, as detailed in our Minimum Standards Reporting Checklist. Information essential to interpreting the data presented should be made available in the figure legends.<br><br>Have you included all the information requested in your manuscript?<br><br>"	The data are not experimental, they come from natural samples. Sampling metadata on the latter are included in Supplementary Table 1.
**Resources**<br><br>A description of all resources used, including antibodies, cell lines, animals and software tools, with enough information to allow them to be uniquely identified, should be included in the Methods section. Authors are strongly encouraged to cite Research Resource Identifiers (RRIDs) for antibodies, model organisms and tools, where possible.<br><br>Have you included the information requested as detailed in our Minimum Standards Reporting Checklist?	Yes
**Availability of data and materials**<br><br>All datasets and code on which the conclusions of the paper rely must be either included in your submission or deposited in publicly available repositories (where available and ethically appropriate), referencing such data using a unique identifier in the references and in the “Availability of Data and Materials” section of your manuscript.	Yes

Have you have met the above requirement as detailed in our Minimum Standards Reporting Checklist?	

Powered by Editorial Manager® and ProduXion Manager® from Aries Systems Corporation

Manuscript
Click here to access/download;Manuscript;Manuscript-final-clean.docx

"UDE DIATOMS in the Wild 2024": A new image dataset of freshwater diatoms for training deep learning models

Authors

Aishwarya Venkataramanan[1,2,3,*], Michael Kloster[4,*], Andrea Burfeid-Castellanos[4], Mimoza Dani[4], Ntambwe A. S. Mayombo[4], Danijela Vidakovic[4,5], Daniel Langenkämper[6], Mingkun Tan[6], Cedric Pradalier[2], Tim Nattkemper[6], Martin Laviale[1,3], Bánk Beszteri[4]

Affiliations

[1] Université de Lorraine, CNRS, LIEC, F-57000 Metz, France

[2] Georgia Tech Europe, CNRS IRL 2958, F-57000 Metz, France

[3] LTSER-"Zone Atelier Moselle", F-57000 Metz, France

[4] Phycology Group, Faculty of Biology, University of Duisburg-Essen, Essen, Germany

[5] Institute of Chemistry, Technology and Metallurgy, University of Belgrade, National Institute of the Republic of Serbia, Belgrade, Serbia

[6] Biodata Mining Group, Faculty of Technology, Bielefeld University, Bielefeld, Germany

[*] equal contribution

Corresponding author: michael.kloster@uni-due.de

ORCIDs:

Aishwarya Venkataramanan [0000-0002-6100-0034]; Michael Kloster [0000-0001-9244-4925]; Andrea Burfeid-Castellanos [0000-0002-8556-3157]; Mimoza Dani; Ntambwe Albert Serge Mayombo [0000-0001-6418-1419]; Danijela Vidakovic [0000-0002-8696-7743]; Daniel

Langenkämper [0000-0003-1857-5040]; Mingkun Tan [0000-0002-5997-9087]; Cedric Pradalier [0000-0002-1746-2733]; Tim Nattkemper [0000-0002-7986-1158]; Martin Laviale [0000-0002-9719-7158]; Bank Beszteri [0000-0002-6852-1588]

Abstract

Background: Diatoms are microalgae with finely ornamented microscopic silica shells. Their taxonomic identification by light microscopy is routinely used as part of community ecological research as well as ecological status assessment of aquatic ecosystems, and a need for digitalisation of these methods has long been recognized. Alongside their high taxonomic and morphological diversity, several other factors make diatoms highly challenging for deep learning-based identification using light microscopy images. These include a) an unusually high intra-class variability combined with small between-class differences; b) a rather different visual appearance of specimens depending on their orientation on the microscope slide; and c) the limited availability of diatom experts for accurate taxonomic annotation.

Findings: We present the largest diatom image dataset thus far, aimed at facilitating the application and benchmarking of innovative deep learning methods to the diatom identification problem on realistic research data, "UDE DIATOMS in the Wild 2024". The dataset contains 83,570 images of 611 diatom taxa, 101 of which are represented by at least 100 examples, and 144 by at least 50 examples each. We showcase this dataset in two innovative analyses that address individual aspects of the above challenges using subclustering to deal with visually heterogeneous classes, out-of-distribution sample detection and semi-supervised learning.

Conclusions: The problem of image-based identification of diatoms is both important for environmental research, and challenging from the machine learning perspective. By making available the so far largest image data set, accompanied by innovative analyses, this contribution will facilitate addressing these points by the scientific community.

Keywords

Diatom, light microscopy, digital imaging, slide scanning, aquatic ecology, deep learning, out-of-distribution detection, semi-supervised learning

Data description

Context

Diatoms, in systematics mostly referred to as Bacillariophyta [1], though recently also as Diatomea [2], a subgroup of the Stramenopiles under the supergroup TSAR [3], are an ecologically important group of single-celled, chlorophyll-*a* and -*c* containing microalgae. One of their main characteristic cellular features is their production of peculiarly shaped and patterned cell walls, termed frustules, that are composed of approximately 90 % amorphous silica [4]. Diatoms are ubiquitous and often abundant in diverse aquatic habitats [5, 6] and contribute substantially to numerous important ecosystem functions and biogeochemical cycles [7, 8]. There are an estimated 10,000 to 30,000 described species of diatoms, with many more waiting to be discovered [9, 10]. Although morphology alone is often insufficient to diagnose diatom species [11], the morphologically recognizable diversity of diatoms is probably larger than that of any other protistan group. This morphological diversity has been the basis of a widespread use of these organisms as ecological and paleo-ecological indicators both in basic and applied research as well as in regulatory biomonitoring [12-15].

A need for a digital transformation of these light microscopic and manual identification methods has long been recognized based on numerous factors. For one, the number of taxonomic experts capable of diatom identification is low and can become a

limiting factor when aiming to scale up the spatial-temporal coverage of ecological and biodiversity monitoring [16]. More fundamentally, digital image-based methods have the potential to enable an improved consistency, reproducibility and objectivity of diatom analysis when compared to identifications performed by human experts directly on a microscope [17, 18]. Experiences indicate that inconsistencies in diatom identification and enumeration can be substantial between different analysts [19-21], which has also been observed for other organismal groups [22, 23]. Over 20 years ago, the ADIAC project developed fundamental approaches for digital imaging and identification [24]. More than ever, we now need standardized, digital imaging methods combined with digitally supported taxonomic identification in order to have objective, reproducible, and comparable taxonomic data for rapid processing of large numbers of samples.

With improving possibilities of digital image acquisition and analysis, methods combining medium- to large-scale image data collection with deep neural networks have recently spread rapidly in biodiversity research [25-27], including in the aquatic and microscopic realm [28, 29]. In the case of diatoms, though not yet broadly applied, slide scanning microscopy now provides a possibility of large-scale digital image acquisition suitable for the standard type of diatom preparations [18, 30-34].

High resolution / high numerical aperture objectives required for diatom analysis offer only a very limited focal depth, so that usually either the valve shape or the valve ornamentation can be seen clearly at a time. Yet, for taxonomic identification often both of them need to be considered. In manual microscopy, this predicament is solved by focusing up and down through the three-dimensional structure of a valve until all relevant features have been observed. In previously published diatom image datasets, a single focal plane was preselected by a human expert to expose the most relevant features for each specimen,

depending on valve orientation and species. Such a manual approach is not an option in an automated high-throughput processing pipeline, and the problem of finding the optimal focal plane for taxonomic identification of each diatom specimen automatically has not been solved yet. However, automated slide scanning allows to image a multitude of focal planes and compress their visual information into a single image by focus stacking. This way, all relevant features are contained within a single image, which massively simplifies downstream processing and analysis.

A range of studies have tested the application of deep learning (DL) models for diatom object detection [35-40], counting [41], segmentation [42, 43], and classification [30, 44-49]. Here the term "classification" is used in the machine learning sense, i.e., referring to machine learning models with a categorical target variable; in a biological terminology it usually addresses taxonomic identification. Diatom localization (using object detection or segmentation models) can now be performed with a high accuracy, even on gigapixel-sized slide scans sometimes termed "virtual slides" [35, 43, 50]; the classification problem (taxon identification), however, remains highly challenging.

Several factors make the diatom classification problem particularly challenging from the machine learning or computer vision perspective. The high number of observed species is a challenge by itself: even when focusing on a local or regional flora, the number of diatom species often lies in the hundreds. In geographically more extended settings, the number of species can quickly reach thousands [51]. According to published experiences, between 50-100 examples (ideally, more) per taxon are required for deep learning model training to reach satisfying classifier performances [45, 46]. Collecting and annotating so many images using a manual approach (as done so far in most diatom deep learning studies) is highly time-consuming. The problem is exacerbated by the uneven distribution of taxa, leading to

most species being encountered comparatively rarely. This is not a peculiarity of diatoms, but results from the general ecological phenomenon often termed hollow abundance distributions [52, 53]. From the machine learning perspective, this leads to a class imbalance problem [54-56]. On the practical side, a consequence is that collecting sufficient examples for rare taxa can take orders of magnitude more effort than capturing common taxa.

A further challenging aspect of image-based diatom identification can be summarized as a generally high intra-class (intraspecific) variability often paired with very minute between-class (interspecific) differences (Figure 1, Figure 2). This is connected to two features of the biology of diatoms. First, the diatom life cycle entails a cyclic alteration of size reduction (accompanying vegetative divisions) with size restitution commonly linked with sexual reproduction [57-59]. In taxa with elongated shapes, size diminution is disproportionately faster in the apical (length) than the transapical (width) direction, leading to substantial shape changes during the life cycle (Figure 2a). Second, environmental effects such as nutrient availability, salinity or temperature can also lead to morphological variations (ecomorphologies, Figure 2b; phenotypic plasticity, Figure 2c). It is common in elongated-shaped diatoms that similar-sized representatives of different closely related species appear visually more similar to each other than to differently sized specimens of the same species [60, 61]. Furthermore, the geometric properties of diatom frustules lead to a further complication in that diatom cells or valves are mostly encountered on microscopic slides in certain viewing angles, mostly in valvar (looking directly onto the valve surface) and/or pleural (looking at the girdle bands) view, with intermediate (tilted) orientations missing or rare (Figure 2d). This leads to two visually distinct projections representing a single taxon in the light microscopic view. Human analysts learn to interpret and link these views with experience. However, these distinctly different visual appearances probably present a

substantial challenge for typical deep learning models by possibly leading to within-class discontinuities in feature space. A further difficulty for algorithms and human analysts alike are taxonomically difficult groups (sometimes referred to as species complexes or *sensu lato* groups), which means that very similar taxa with partially still unresolved taxonomic status show high variability but also intermediate morphologies (Figure 2e). The existence of heterovalvar diatoms, those that have two valves with differences in the ornamentations, can also lead to distinct visual appearances within a taxon (Figure 2f).

Routine diatom preparations often also contain disturbing background particles such as sediment, clay, small diatom fragments, sometimes remains of other organisms e.g. sponge needles etc. (Figure 3). Although careful adjustments during slide preparation can help reduce overlaps of diatom frustules / valves with such disturbing particles and with each other, such adjustments are rarely performed systematically during routine diatom analysis. This often leads to a situation where diatom frustules touch or overlap with disturbing non-diatom particles or other diatoms, making the visual recognition of taxa more challenging. Even though these issues are very common, with very few exceptions [43] they are not covered by the currently available diatom datasets (Table 1). Instead of pre-selecting "clean" examples, we deliberately included such challenging data to get closer to a real-world situation. Even though we cannot offer a solution to all of these problems within the scope of this work, we would like our image dataset to represent a "real-world" difficulty level, which is important for a realistic assessment of the usability of image analysis methods for routine diatom analysis.

Thus, analyses of light microscopic images of diatoms by deep learning is an urgent need for research of ecology and biodiversity, as well as environmental monitoring. Yet, development of the machine learning and computer vision is a challenge. One main obstacle currently

slowing the development of the field is the scarcity of datasets that are suitable for training and comparing deep learning models. There are very few publicly available diatom image datasets, and the available ones are mostly too small for training deep learning models. The first published taxonomically annotated light microscopic image dataset addressing a machine learning utilization before the deep learning era came from the ADIAC project [24, 62], and contains ca. 3,400 images representing 328 species. A substantial image dataset known as Aqualitas was assembled a few years ago [45, 61, 63, 64], covering 100 diatom taxa with about 100 images each. However, the Aqualitas images seem to depict isolated diatom cells, imaged at a single focal plane and containing very little or none of the disturbing factors usually observed in routine preparations (see above). So classification may be considered as “too easy” in the context of a non-selective automated imaging workflow. Another dataset was released recently [35], consisting of 9,230 individual images with at least 50 images of 166 diatoms species, which were extracted from pdf versions of publicly available taxonomic atlases [65-67], as well as ca. 600 images of real debris. Another recent study [68] collated images from diatoms.org [69], an online identification aid illustrated by thousands of diatom images, nevertheless still with a relatively low number of examples per species. One dataset containing slightly over 3,300 images of 10 taxa [70] and another one that contains images and segmentation masks for 3,027 diatoms from 68 species [71, 72] are available in public repositories. Two more taxonomically annotated image datasets have been published by Burfeid Castellanos et al. from a manual digital diatom analysis workflow [18]. These contain 18,441 images of 120 species [73, 74] and 8,858 images of 161 species [75], respectively, averaging to 153 and 55 examples per species, although both datasets are imbalanced. The latter two datasets were not explicitly aimed at machine learning utilization, and were thus not formatted in a way that would be immediately usable in such a

context, but could, in principle, also be useful for this purpose. Nevertheless, most published datasets are not ideally suited for deep learning experiments because they are relatively small; Table 1 summarizes basic information on currently available diatom image datasets. We note that for planktonic organisms, a much larger collection of datasets is publicly available, these were recently reviewed [76].

In this paper, we present a novel light microscopic image dataset of freshwater diatoms that a) is substantially larger than those previously available; b) was obtained using a reproducible slide scanning and annotation workflow following standard counting procedures for water quality monitoring [77]; c) reflects a “real-life” challenge (i.e., it is not limited to manually selected examples that might be biased towards well recognizable diatoms without e.g. overlapping debris), d) covers the shape as well as the ornamentation of valves / frustules in the same image due to focus stacking, and e) is publicly available to support customizing and benchmarking deep learning models to this field of application. To highlight the challenging nature of this dataset, as well as to propose possible avenues to address some of these challenges, we provide two deep learning experiments, one addressing out-of-distribution detection and modelling within-class heterogeneity, another one leveraging semi-supervised learning to alleviate the need for voluminous labelled training data.

Methods

Sampling and preparation

A total of 318 samples of freshwater diatoms were gathered from 15 different localities following standardized methodology[78], by scraping the biofilm from submerged stones selecting an area of approximately 20 cm². A total of five stones per sampling site

were sampled and pooled together. When no stones were available, either previously submerged artificial substrates, woody surfaces (epidendron), submerged plants (epiphyton) or sand (epipsammon) were sampled (Appendix 1). The samples were then preserved with molecular grade ethanol to a final concentration of 75 % and stored at -20 °C.

Diatom preparation followed the hot H_2O_2-HCl digestion method[79]. During five wash-cycles, the samples were centrifuged at 464 g for four min, followed by discarding the supernatant and refilling with deionised water. The resulting “clean” sample was oxidized by first treating with 30 % hydrogen peroxide (H_2O_2), heating up to 90 °C for 3-4 h. After the H_2O_2 had evaporated, the samples were left to cool down. Subsequently, 37 % hydrogen chloride (HCl) was added to the cooled samples to dissolve the remaining organic matter and carbonates. Finally, after the reaction stopped, the samples were again washed to avoid acid corrosion through prolonged exposure, following the same procedure as during the pre-wash cycle. After seven cycles, the sample was suspended in 1 ml deionised water plus 2-3 drops of ethanol or glycerine.

After adding a small amount of 10 % ammonium chloride solution to the suspension, it was spread onto coverslips and dried on a heating plate at 350 °C. The dried sample on the coverslip was embedded in Naphrax artificial resin with a nominal refractive index of 1.72 (Thorns Biologie Bedarf, Deggendorf, Germany). The slides were left to harden for one to two weeks before scanning.

Imaging by slide scanning

The slide preparations were digitized with a VS200 slide scanning microscope (Olympus Europa SE & Co. KG, Hamburg, Germany) in bright-field mode using an UPLXAPO60XO 60x/1.42 oil immersion objective. Depending on the preparation’s material density, usually 16 or 25 mm² per slide were scanned in the form of a contiguous rectangular

area. To cover the thickness of the sample, mostly 40 – 85 different focal planes were imaged at a distance of 0.28 µm each; this corresponds to half of the objective's focal depth and warrants that each detail of the valve ornamentation is captured within at least one focal plane. However, due to excessive digital filtering, the VS200 integrated focus stacking tends to suppress fine repetitive structures, which are often essential for diatom identification. To overcome this limitation, we implemented our own post-processing pipeline utilizing Helicon Focus[80] for focus stacking, the ImageJ plugin "MIST"[81] for position registration of adjacent field of view images, and the ImageJ plugin "Grid / collection stitching" [82] for stitching them. Since processed diatom silica does not provide colour information, we reduced the 24-bit RGB data to 8-bit grayscale / intensity. A typical slide scan resulted in several gigapixels of image data, divided into subsections of less than two gigabyte uncompressed image data, to avoid restrictions of typical image processing tools and libraries. We refer to such images as "virtual slide images".

Annotation

Diatoms were annotated using the BIIGLE 2.0 [83] web tool by four annotators (each image was annotated by one of them). Most of the diatom annotations followed the "traditional" microscopy-based workflow as close as possible, screening through a contiguous rectangular area of the virtual slide image. A few samples were processed using random sampling or the so-called lawnmower mode. The latter guides the user over the virtual slide image in a similar serpentine pattern as used during manual counting [83]. As annotation shapes, rectangular bounding boxes, circles or polygons roughly outlining the diatom were used. Most annotation shapes were labelled by the specimen's taxonomic name at the species level, some only at genus or down to subspecies level. Taxonomic

identification followed standard methodology [77], and was undertaken using general and specific literature [65, 84, 85].

After the identification of at least 400 valves per sample was completed, quality control and consistency checking were executed in a taxon-by-taxon manner with the label review grid overview (LARGO) feature of BIIGLE 2.0 [18].

Dataset preparation

The annotations were extracted from BIIGLE via the BIIGLE REST API. Subsequently, for each annotation, relevant information was converted into CSV format, and corresponding cutouts from the gigapixel slide scans were generated. Throughout processing, image data was stored in lossless file formats to prevent introducing compression artefacts. We named this dataset “UDE DIATOMS in the Wild 2024” (University of Duisburg-Essen – Digital annotated open-source microscope slide scans from real-world samples, version of 2024).

Data visualization using dimensionality reduction

To demonstrate the dataset's challenges and to support rendering a mental model of the data distribution, we showcase a 2D scatterplot in Figure 4, depicting the ten most abundant species. To generate this figure, we computed a high-dimensional feature for each cutout using a ViT-L/16 vision transformer model [ViT-L/16, 86], and projected this feature into a two-dimensional data space using *t*-distributed stochastic neighbor embedding [87] (t-SNE). The embedded data was visualized using a scatterplot, where species membership is indicated by the colours used. Each data point therefore depicts one cutout. In Supplement Figure 1, an interactive 3D version is available, allowing the visualization of all 144 species

represented by at least 50 examples, with the ability to hide or display certain species interactively.

Dataset description

All the samples processed for this dataset were taken in continental rivers, streams and lakes, the salinity of the habitats varied from freshwater to saline. Supplement Table 1 contains the sampling metadata for the 319 virtual slides from which the image cutouts were generated. Table 2 contains information on the annotations that are included in the dataset as comma-separated fields (with strings quoted). The image cutouts are based on very roughly, manually annotated object shapes or rotated bounding rectangles, which usually include a substantial margin around the objects, and are provided as 8-bit grayscale / intensity PNG files with a uniform resolution of 0.09 µm/pixel. The dataset contains 83,570 images of 611 diatom taxa. 74,410 of these images were identified at the species level to 542 species (Supplement Table 2), the rest to 69 genera. 101 species are represented by at least 100 examples each (67,594 images in total), 144 species by at least 50 examples (70,567 images in total), and 196 by at least 25 examples (72,405 images in total). The abundance distribution is highly skewed, i.e., the dataset is strongly imbalanced, as typical for non-selectively collected biodiversity data (Figure 5).

Re-use potential

We present two deep learning experiments, each addressing particular challenges of deep learning as applied to diatom analysis. The first experiment uses a deep learning approach to handle the detection of out-of-distribution samples and explicitly models intra-class heterogeneity. Out-of-distribution detection should pinpoint specimens of taxa not present in the training set. Modelling within-class heterogeneity can help to address the

distinct visual appearance of valves lying in different orientations relative to the microscope view. The second experiment investigates the potential of semi-supervised learning (SSL) to alleviate the need for human expertise to annotate image collections. Here, SSL utilizes unlabelled image data to learn better feature representations. The results are compared to a study conducted with a vision transformer model.

Deep learning experiment 1: out-of-distribution sample detection

In this experiment, we addressed the problem of detecting out of distribution (OOD) samples. Deep learning classifiers often exhibit a tendency to make overconfident predictions when confronted with OOD data, erroneously classifying them as belonging to one of the classes within their training data, resulting in unreliable model outputs [88, 89]. This corresponds to a situation where a model encounters a species not represented in its training set. Instead of classifying such examples into the next best species available, it would be preferable to recognize such cases as novelties. A closely related problem is the preference of many diatom species to settle mostly at specific viewing angles on the slide (Figure 2d) and only rarely in intermediate orientations. This leads to a discontinuous feature space, where models would need to learn to classify visually rather distinct appearances into one and the same class. This can be addressed by considering distinct views as OOD samples for other views and therefore splitting a class into visually more homogeneous clusters, which is accomplished by moving such OOD examples into appropriate own classes. In general, our OOD-detection approach could enhance the reliability and safety of deep learning classifiers when facing data that deviates from their training distribution, but also in cases when single classes are represented by visually distinctly different clusters of images.

For the experiments, we considered two subsets of the data. Dataset D25 included 196 classes (species) represented by at least 25 examples (individuals) as in-distribution dataset,

with the images from the remaining 346 classes used as OOD data. Dataset D50 included 144 classes represented by at least 50 examples, with the images from the remaining 398 classes being used as OOD data. For both D25 and D50, 70% of the images from the in-distribution datasets were used for training, 20% for validation and 10% for testing.

An EfficientNet network, pretrained on ImageNet [96] was trained using our method called MAPLE (MAhalanobis distance based uncertainty Prediction for reLiablE classification[90]) illustrated in Figure 6. To address high intra-class variances due to, for instance, different viewpoints from which the images were acquired, we use X-Means clustering[91] to break down classes into multiple clusters, each of which contains images clustering together in the feature space of representations learned by the network. These clusters are then treated as if they were different classes during the training process.

The triplet loss [92] during our training serves to bring similar samples from the same class closer together and push them farther away from samples in other classes. This approach assists the model in distinguishing between diatoms that look similar but belong to different classes.

As baseline for comparison, the standard ImageNet-pretrained EfficientNet model trained using cross-entropy loss was used. We refer to this baseline as the deterministic counterpart of MAPLE in the results below.

Accuracy and F1-score (Table 3) were used to assess classification performance of the models (in the case of MAPLE, on in-distribution data). In addition, we used AUROC (area under the receiver operating characteristic curve) and AUPR (area under the precision-recall curve) scores for evaluation of OOD sample detection in the experiment, following common practice in the OOD literature [93-95]. The AUROC metric measures the model's ability to distinguish between in-distribution and out-of-distribution instances across various decision

threshold settings. Similarly, the AUPR metric emphasizes the model's ability to perform well in situations with class imbalance. In the case of the deterministic baseline, we used the probabilities from the softmax values, and in the case of MAPLE, the probability derived from Mahalanobis distance.

Although accuracy of the deterministic model was marginally better, MAPLE achieved a higher AUROC and AUPR score compared to the deterministic classifier for both the D25 and the D50 datasets (Table 3 and Figure 7). This outcome signifies that MAPLE demonstrates superior performance in terms of OOD sample detection.

Figure 8 illustrates subclusters found within individual species by MAPLE, which often correspond to morphologically interpretable visual differences: for instance, pleural vs. valvar views (Figure 8a-b) in *Achnanthidium atomoides*, or single vs. both valves in *Amphora pediculus* (Figure 8c-d). In some cases, e.g. *Fragilaria pectinalis*, different subclusters contain what seem to represent different phases of a size reduction series (Figure 8e-f). It is unclear if this might be an artefact of having sampled two relatively distinct parts of a morphological continuum, or caused by the fact that visual variation along the size axis is so much larger than in other directions. These aspects merit further investigation.

Deep learning experiment 2: semi-supervised learning

In our second set of experiments, we examined the impact of semi-supervised learning (SSL) on diatom classification. SSL is a methodology to improve classification performance by using unlabelled data [96-101]. The basic idea is that prior to training the classifier in the usual supervised way, a so-called pretext task is learned. This pretext task may be, e.g. as in our case, to recreate parts of the image that have previously been randomly masked (i.e. restore the full image from a version where portions of it had been deleted). For these tasks no label information is necessary, that is why it is called semi-supervised learning. During the

pretext task, the algorithm learns a representation of the data in general. These representations are technically the same as a pre-trained model, i.e. weights that are loaded by the algorithm, just like the usually used ImageNet pretrained models. In the SSL experiments we utilised the 144 classes from the UDE Diatoms in the Wild 2024 data that contained a minimum of 50 examples (DS50 dataset, as in experiment 1). This dataset was divided into a training set called D^{t} (80%), and a test set called $\mathrm{D}^{\mathrm{test}}$ (20%). Furthermore, we randomly selected 10% of the data from each class in D^{t} as the reduced training subset, named $\mathrm{D}^{\mathrm{t}}_{0.1}$, to simulate a scenario where training data was limited and to study the impact of SSL in this case. The structure of the datasets is illustrated in Figure 9.

The workflow of our experiments is displayed in Figure 10. To establish a baseline, we used a ResNet50 (hereafter referred to as RN for brevity) convolutional neural network and a ViT-Large (ViT-L/16, hereafter referred to as ViT for brevity) [ViT-L/16, 86] vision transformer model which had been pre-trained on ImageNet[92] data, fine-tuned it on D^{t}, and evaluated it on $\mathrm{D}^{\mathrm{test}}$. These experiments are referred to as $\mathrm{RN}_{\mathrm{D}^{\mathrm{t}}}$ and $\mathrm{ViT}_{\mathrm{D}^{\mathrm{t}}}$, respectively. We conducted identical experiments, utilising the smaller $\mathrm{D}^{\mathrm{t}}_{0.1}$ training data subset, referring to them as $\mathrm{RN}_{\mathrm{D}^{\mathrm{t}}_{0.1}}$ and $\mathrm{ViT}_{\mathrm{D}^{\mathrm{t}}_{0.1}}$, respectively.

To compare a semi-supervised approach with the ViT baseline, we employed a masked auto-encoder (MAE) [96] using the same backend ViT. This MAE had already been pre-trained using SSL on ImageNet data, and we fine-tuned it on D^{t}. In this case non-domain data was used for SSL training, but the fine-tuning was done on in-domain data. These experiments are denoted as $\mathrm{MAE}_{\mathrm{D}^{\mathrm{t}}}$ and $\mathrm{MAE}_{\mathrm{D}^{\mathrm{t}}_{0.1}}$.

The results of the experiment showed that network performance benefited from SSL, whether fine-tuned with the whole labelled dataset D^{t} or with only 10% of the labelled data $\mathrm{D}^{\mathrm{t}}_{0.1}$, as measured both by macro- and micro-averaged metrics [102](Table 4).

Conclusion from deep learning experiments

Our results reached substantially lower accuracies, in comparison to deep learning experiments previously applied to diatom data [45]. We attribute this to our non-selective imaging method, which impacts the specimen and image quality as well as the background homogeneity and also has an effect on the intra- and inter-class variations of features, all of which probably make our “UDE Diatoms in the Wild 2024” dataset more challenging, but also reflective of a typical use case. As discussed in the introduction, this is by design: we think it is important to apply and test image analysis methods on types of image data that can be produced by high throughput imaging methods, as opposed to manual selection and focusing by a human expert.

Beyond its relevance to diatom analysis and more broadly to biodiversity and environmental research, this dataset is demanding also from a general computer vision point of view. Unlike previously available “clean” datasets, which are typically used as benchmarks in the computer vision community, this dataset contains several of the problems typically encountered when dealing with real-life datasets. This includes a class imbalance, resulting in a long-tailed distribution of the images for classification. Such class imbalances pose difficulties for machine learning approaches as the overrepresented classes have a stronger influence on the acquired model. Additionally, the dataset exhibits high levels of inter-class similarity and intra-class variance due to the special visual features of diatoms outlined in the Introduction. Moreover, the presence of occlusions (diatoms being partly concealed by overlapping objects) within the dataset adds another layer of complexity. Dealing with occlusions requires robust feature extraction and recognition capabilities to effectively discern obscured objects. Some of these problems are not unique to diatom classification, but are general problems investigated by computer vision for decades now. Given these

listed observations, this dataset can be seen as a valuable resource not only for diatom research, but also for addressing some more generic challenges in computer vision .

We would also argue that for applicability of digital imaging and identification methods for routine diatom community characterization or for instance water quality monitoring, intelligent combinations of advanced models (going beyond simple supervised classification, like our baseline models) will be necessary. For instance, as experiment 2 shows, semi-supervised learning has a potential to alleviate the need for labelled training data; whereas out-of-distribution detection, as possible in MAPLE (experiment 1), has the potential to address detecting taxa not represented in a training set, another practically relevant aspect of real life analyses. How to best combine these strengths to a best overall digital diatom community analysis workflow, is currently an open question.

Data availability

Supporting data are available from the GigaScience database GigaDB [103], with the full dataset deposited in Zenodo [104]. For easy practical application, subsets containing training, validation and test data (60%:20%:20% split) of species represented by at least 25, 50 or 100 specimens each, and stored in the simple torchvision DatasetFolder-dataset structure with one folder per species, are available from Kaggle [105].

Availability of source code and requirements

Project name: UDE Diatoms in the Wild – experiment 1: MAPLE out-of-distribution detection

Project home page: https://github.com/vaishwarya96/maple-ude

Operating system(s): LINUX

Programming language: Python 3

Other requirements: described in the GitHub repository

License: MIT License

Project name: UDE Diatoms in the Wild – experiment 2: semi-supervised learning

Project home page: https://github.com/mtan-unibie/GIGASci

Operating system(s): LINUX

Programming language: Python 3

Other requirements: described in the GitHub repository

License: MIT License

Funding

M.K. and D.L. were funded by the Deutsche Forschungsgemeinschaft (DFG, German Research Foundation; project number: 463395318). M.D., A.B.C., N.A.S.M. were partially funded by the Collaborative Research Centre 1439 RESIST (Multilevel Response to Stressor Increase and Decrease in Stream Ecosystems; www.sfb-resist.de) funded by the DFG (CRC 1439/1, project number: 426547801). ABC was also partially supported by the EU through the PRIMA project (INWAT 201980E121), which was sponsored by the German Federal Ministry of Education and Research. Funding for D.V. was provided by the Humboldt Foundation. The PhD scholarship for A.V. was funded by ANR, France (ANR-20-THIA-0010) and Région Grand-Est, France. Additional financial support was provided by CNRS, France (ZAM LTSER Moselle) and Horizon Europe (iMagine – Grant agreement ID: 101058625). This

publication was supported by the University of Duisburg-Essen Open Access Publication Fund.

Author contributions

B.B., M.K., D.L., C.P., T.N., and M.L. designed the study. A.B.C., M.D., N.A.S.M. and D.V. annotated the images. M.K. performed the image acquisition, handling and data curation. A.V., D.L. and M.T. performed the illustrating analyses. B.B., A.V., M.K., A.B.C., D.L. and M.T. drafted the manuscript. All authors contributed to writing the manuscript.

Competing interests

The authors declare no competing interests.

References

1. Mann DG, Crawford RM and Round FE. Bacillariophyta. In: Handbook of the Protists. 2016:1-62. doi:10.1007/978-3-319-32669-6_29-1.
2. Adl SM, Bass D, Lane CE, Lukeš J, Schoch CL, Smirnov A, et al. Revisions to the classification, nomenclature, and diversity of eukaryotes. Journal of Eukaryotic Microbiology. 2019;66 1:4-119. doi:10.1111/jeu.12691.
3. Burki F, Roger AJ, Brown MW and Simpson AG. The new tree of eukaryotes. Trends Ecol Evol. 2020;35 1:43-55.
4. Kröger N and Poulsen N. Diatoms-From Cell Wall Biogenesis to Nanotechnology. Annual Review of Genetics. 2008;42 1:83-107. doi:10.1146/annurev.genet.41.110306.130109.
5. Burliga AL and Kociolek JP. Diatoms (Bacillariophyta) in Rivers. In: River Algae. 2016:93-128. doi:10.1007/978-3-319-31984-1_5.
6. Tomas CR. Identifying marine phytoplankton. Elsevier; 1997.
7. Granum E, Raven JA and Leegood RC. How do marine diatoms fix 10 billion tonnes of inorganic carbon per year? Canadian Journal of Botany. 2005;83 7:898-908. doi:10.1139/b05-077.
8. Nelson DM, Tréguer P, Brzezinski MA, Leynaert A and Quéguiner B. Production and dissolution of biogenic silica in the ocean: revised global estimates, comparison with regional data and relationship to biogenic sedimentation. Global biogeochemical cycles. 1995;9 3:359-72. doi:10.1029/95GB01070.
9. Mann DG and Vanormelingen P. An Inordinate Fondness? The Number, Distributions, and Origins of Diatom Species. Journal of Eukaryotic Microbiology. 2013;60 4:414-20. doi:10.1111/jeu.12047.

10. Guiry MD. How many species of algae are there? Journal of Phycology. 2012;48 5:1057-63. doi:10.1111/j.1529-8817.2012.01222.x.
11. Alverson AJ. Molecular Systematics and the Diatom Species. Protist. 2008;159 3:339-53. doi:10.1016/j.protis.2008.04.001.
12. Smol JP and Stoermer EF. The diatoms: applications for the environmental and earth sciences. Cambridge University Press; 2010.
13. Lobo EA, Heinrich CG, Schuch M, Wetzel CE and Ector L. Diatoms as Bioindicators in Rivers. In: River Algae. 2016:245-71. doi:10.1007/978-3-319-31984-1_11.
14. Potapova M and Charles DF. Diatom metrics for monitoring eutrophication in rivers of the United States. Ecological indicators. 2007;7 1:48-70. doi:10.1016/j.ecolind.2005.10.001.
15. Feio MJ, Hughes RM, Callisto M, Nichols SJ, Odume ON, Quintella BR, et al. The Biological Assessment and Rehabilitation of the World’s Rivers: An Overview. Water. 2021;13 3:371. doi:10.3390/w13030371.
16. Carraro L, Mächler E, Wüthrich R and Altermatt F. Environmental DNA allows upscaling spatial patterns of biodiversity in freshwater ecosystems. Nature Communications. 2020;11 1 doi:10.1038/s41467-020-17337-8.
17. Cristóbal G, Blanco S and Bueno G. Overview: Antecedents, Motivation and Necessity. In: Modern Trends in Diatom Identification. 2020:3-10. doi:10.1007/978-3-030-39212-3_1.
18. Burfeid-Castellanos AM, Kloster M, Beszteri S, Postel U, Spyra M, Zurowietz M, et al. A Digital Light Microscopic Method for Diatom Surveys Using Embedded Acid-Cleaned Samples. Water. 2022;14 20:3332.
19. Kelly MG, Bayer MM, Hürlimann J and Telford RJ. Human error and quality assurance in diatom analysis. In: Automatic diatom identification. 2002:75-91. doi:10.1142/9789812777867_0005.
20. Kahlert M, Kelly M, Albert R-L, Almeida SFP, Bešta T, Blanco S, et al. Identification versus counting protocols as sources of uncertainty in diatom-based ecological status assessments. Hydrobiologia. 2012;695 1:109-24. doi:10.1007/s10750-012-1115-z.
21. Beszteri B, Allen C, Almandoz GO, Armand L, Barcena MÁ, Cantzler H, et al. Quantitative comparison of taxa and taxon concepts in the diatom genus *Fragilariopsis*: a case study on using slide scanning, multiexpert image annotation, and image analysis in taxonomy. Journal of Phycology. 2018;54 5:703-19. doi:10.1111/jpy.12767.
22. Culverhouse P, Williams R, Reguera B, Herry V and González-Gil S. Do experts make mistakes? A comparison of human and machine identification of dinoflagellates. Marine Ecology Progress Series. 2003;247:17-25. doi:10.3354/meps247017.
23. MacLeod N, Benfield M and Culverhouse P. Time to automate identification. Nature. 2010;467 7312:154-5.
24. du Buf H and Bayer MM. Automatic diatom identification. Singapore: World Scientific; 2002.
25. Christin S, Hervet É and Lecomte N. Applications for deep learning in ecology. Methods in Ecology and Evolution. 2019;10 10:1632-44. doi:https://doi.org/10.1111/2041-210X.13256.
26. Borowiec ML, Dikow RB, Frandsen PB, McKeeken A, Valentini G and White AE. Deep learning as a tool for ecology and evolution. Methods in Ecology and Evolution. 2022;13 8:1640-60.

27. Goodwin M, Halvorsen KT, Jiao L, Knausgård KM, Martin AH, Moyano M, et al. Unlocking the potential of deep learning for marine ecology: overview, applications, and outlook. Ices J Mar Sci. 2022;79 2:319-36.
28. Madkour DM, Shapiai MI, Mohamad SE, Aly HH, Ismail ZH and Ibrahim MZ. A Systematic Review of Deep Learning Microalgae Classification and Detection. IEEE Access. 2023:1-. doi:10.1109/access.2023.3280410.
29. Orenstein EC, Ayata S-D, Maps F, Becker ÉC, Benedetti F, Biard T, et al. Machine learning techniques to characterize functional traits of plankton from image data. Limnology and Oceanography. 2022;67 8:1647-69. doi:https://doi.org/10.1002/lno.12101.
30. Zhou Y, Zhang J, Huang J, Deng K, Zhang J, Qin Z, et al. Digital whole-slide image analysis for automated diatom test in forensic cases of drowning using a convolutional neural network algorithm. Forensic Sci Int. 2019;302:109922.
31. Kloster M, Esper O, Kauer G and Beszteri B. Large-Scale Permanent Slide Imaging and Image Analysis for Diatom Morphometrics. Applied Sciences. 2017;7 4:330. doi:10.3390/app7040330.
32. Sánchez C, Ruiz-Santaquiteria Alegre J, Espinosa Aranda JL and Salido J. Automatization Techniques. Slide Scanning. In: Modern Trends in Diatom Identification. 2020:113-31. doi:10.1007/978-3-030-39212-3_7.
33. Lu Q, Liu G, Xiao C, Hu C, Zhang S, Xu RX, et al. A modular, open-source, slide-scanning microscope for diagnostic applications in resource-constrained settings. Plos One. 2018;13 3:e0194063.
34. Salido J, Sánchez C, Ruiz-Santaquiteria J, Cristóbal G, Blanco S and Bueno G. A Low-Cost Automated Digital Microscopy Platform for Automatic Identification of Diatoms. Applied Sciences. 2020;10 17:6033.
35. Venkataramanan A, Faure-Giovagnoli P, Regan C, Heudre D, Figus C, Usseglio-Polatera P, et al. Usefulness of synthetic datasets for diatom automatic detection using a deep-learning approach. Engineering Applications of Artificial Intelligence. 2023;117:105594. doi:https://doi.org/10.1016/j.engappai.2022.105594.
36. Yu W, Xiang Q, Hu Y, Du Y, Kang X, Zheng D, et al. An improved automated diatom detection method based on YOLOv5 framework and its preliminary study for taxonomy recognition in the forensic diatom test. Frontiers in Microbiology. 2022;13:963059. doi:10.3389/fmicb.2022.963059.
37. Yu W, Xue Y, Knoops R, Yu D, Balmashnova E, Kang X, et al. Automated diatom searching in the digital scanning electron microscopy images of drowning cases using the deep neural networks. International journal of legal medicine. 2021;135 2:497-508. doi:10.1007/s00414-020-02392-z.
38. Deng J, Guo W, Zhao Y, Liu J, Lai R, Gu G, et al. Identification of diatom taxonomy by a combination of region-based full convolutional network, online hard example mining, and shape priors of diatoms. International Journal of Legal Medicine. 2021;135:2519-30.
39. Gong S, Wu K, Xia Z, Ran L, Gu C, Lu C, et al. An Oriented Object Detector towards Diatoms. 2023 International Joint Conference on Neural Networks (IJCNN). 2023:1-8. doi:10.1109/IJCNN54540.2023.10191878.
40. Zhang J, Vieira DN, Cheng Q, Zhu Y, Deng K, Zhang J, et al. DiatomNet v1. 0: A novel approach for automatic diatom testing for drowning diagnosis in forensically biomedical application. Computer Methods and Programs in Biomedicine. 2023;232:107434. doi:10.1016/j.cmpb.2023.107434.

41. Hou Y, Cui X, Canul-Ku M, Jin S, Hasimoto-Beltran R, Guo Q, et al. ADMorph: A 3D Digital Microfossil Morphology Dataset for Deep Learning. IEEE Access. 2020;8:148744-56. doi:10.1109/access.2020.3016267.
42. Ruiz-Santaquiteria J, Bueno G, Deniz O, Vallez N and Cristobal G. Semantic versus instance segmentation in microscopic algae detection. Engineering Applications of Artificial Intelligence. 2020;87:103271. doi:10.1016/j.engappai.2019.103271.
43. Kloster M, Burfeid-Castellanos AM, Langenkämper D, Nattkemper TW and Beszteri B. Improving deep learning-based segmentation of diatoms in gigapixel-sized virtual slides by object-based tile positioning and object integrity constraint. PLOS ONE. 2023;18 2:e0272103. doi:10.1371/journal.pone.0272103.
44. Lambert D and Green R. Automatic identification of diatom morphology using deep learning. 2020 35th International Conference on Image and Vision Computing New Zealand (IVCNZ). 2020:1-7. doi:10.1109/IVCNZ51579.2020.9290564.
45. Pedraza A, Bueno G, Deniz O, Cristóbal G, Blanco S and Borrego-Ramos M. Automated Diatom Classification (Part B): A Deep Learning Approach. Applied Sciences. 2017;7 5:460.
46. Kloster M, Langenkämper D, Zurowietz M, Beszteri B and Nattkemper TW. Deep learning-based diatom taxonomy on virtual slides. Scientific Reports. 2020;10 1 doi:10.1038/s41598-020-71165-w.
47. Memmolo P, Carcagnì P, Bianco V, Merola F, Goncalves Da Silva Junior A, Garcia Goncalves LM, et al. Learning Diatoms Classification from a Dry Test Slide by Holographic Microscopy. Sensors. 2020;20 21:6353. doi:10.3390/s20216353.
48. Zhang J, Zhou Y, Vieira DN, Cao Y, Deng K, Cheng Q, et al. An efficient method for building a database of diatom populations for drowning site inference using a deep learning algorithm. International Journal of Legal Medicine. 2021;135 3:817-27. doi:10.1007/s00414-020-02497-5.
49. Venkataramanan A, Laviale M, Figus C, Usseglio-Polatera P and Pradalier C. Tackling inter-class similarity and intra-class variance for microscopic image-based classification. International conference on computer vision systems. 2021:93-103. doi:10.1007/978-3-030-87156-7_8.
50. Ruiz-Santaquitaria J, Pedraza A, Sánchez C, Libreros JA, Salido J, Deniz O, et al. Deep Learning Versus Classic Methods for Multi-taxon Diatom Segmentation. Pattern Recognition and Image Analysis: 9th Iberian Conference, IbPRIA 2019, Madrid, Spain, July 1–4, 2019, Proceedings, Part I 9. 2019:342-54. doi:10.1007/978-3-030-31332-6_30.
51. Kociolek JP, You Q, Liu Q, Liu Y and Wang Q. Continental diatom biodiversity discovery and description in China: 1848 through 2019. PhytoKeys. 2020;160:45-97. doi:10.3897/phytokeys.160.54193.
52. Magurran AE and Henderson PA. Explaining the excess of rare species in natural species abundance distributions. Nature. 2003;422 6933:714-6. doi:10.1038/nature01547.
53. McGill BJ, Etienne RS, Gray JS, Alonso D, Anderson MJ, Benecha HK, et al. Species abundance distributions: moving beyond single prediction theories to integration within an ecological framework. Ecology Letters. 2007;10 10:995-1015. doi:10.1111/j.1461-0248.2007.01094.x.
54. Langenkämper D, Van Kevelaer R and Nattkemper TW. Strategies for Tackling the Class Imbalance Problem in Marine Image Classification. In: Pattern Recognition and Information Forensics. 2019:26-36. doi:10.1007/978-3-030-05792-3_3.

55. Haixiang G, Yijing L, Shang J, Mingyun G, Yuanyue H and Bing G. Learning from class-imbalanced data: Review of methods and applications. Expert Systems with Applications. 2017;73:220-39.
56. Johnson JM and Khoshgoftaar TM. Survey on deep learning with class imbalance. Journal of Big Data. 2019;6 1 doi:10.1186/s40537-019-0192-5.
57. Edlund MB and Stoermer EF. Ecological, evolutionary, and systematic significance of diatom life histories. Journal of Phycology. 1997;33 6:897-918. doi:10.1111/j.0022-3646.1997.00897.x.
58. Hense I and Beckmann A. A theoretical investigation of the diatom cell size reduction–restitution cycle. Ecological modelling. 2015;317:66-82. doi:10.1016/j.ecolmodel.2015.09.003.
59. Amato A, Orsini L, D'Alelio D and Montresor M. Life cycle, size reduction patterns, and ultrastructure of the pennate planktonic diatom *Pseudo-nitzschia delicatissima* (Bacillariophyceae). Journal of Phycology. 2005;41 3:542-56. doi:10.1111/j.1529-8817.2005.00080.x.
60. Kloster M, Rigual-Hernández AS, Armand LK, Kauer G, Trull TW and Beszteri B. Temporal changes in size distributions of the Southern Ocean diatom *Fragilariopsis kerguelensis* through high-throughput microscopy of sediment trap samples. Diatom Res. 2019;34 3:133-47. doi:10.1080/0269249X.2019.1626770.
61. Sánchez C, Cristóbal G and Bueno G. Diatom identification including life cycle stages through morphological and texture descriptors. PeerJ. 2019;7:e6770. doi:10.7717/peerj.6770.
62. Automatic Diatom Identification project web page. https://websites.rbge.org.uk/ADIAC/db/adiacdb.htm.
63. Bueno G, Deniz O, Pedraza A, Ruiz-Santaquiteria J, Salido J, Cristóbal G, et al. Automated Diatom Classification (Part A): Handcrafted Feature Approaches. Applied Sciences. 2017;7 8:753.
64. Carlos Sanchez Bueno SB, Gloria Bueno, Maria Borrego-Ramos, Gabriel Cristobal. Aqualitas diatom image database. https://figsharecom/articles/dataset/Aqualitas_Database_full_release_/11728980.
65. Peeters V and Ector L. Atlas des diatomées des cours d'eau du territoire bourguignon. Direction Régionale de l'Environnement, de l'Aménagement et du Logement Bourgogne-Franche-Comté; 2017.
66. Lalanne-Cassou C and Voisin JF. *Atlas des diatomées d'ile de france*. 2013. Direction Régionale et Interdépartmentale de l'Environnement et de l'Energie d'Île-de-France.
67. Bey MY and Ector L. Atlas des diatomées des cours d'eau de la région rhône-alpes. tome 1. Centriques, Monoraphidées. tome 2. Araphidées, Brachyraphidées. tome 3. Naviculacées: Naviculoidées. tome 4. Naviculacées: Naviculoidées. tome 5. Naviculacées: Cymbelloidées, Gomphonematoidées. tome 6. Bacillariacées, Rhopalodiacées, Surirellacées. Direction Régionale de l'Environnement, de l'Aménagement et du Logement Rhône-Alpes; 013. .
68. Pu S, Zhang F, Shu Y and Fu W. Microscopic image recognition of diatoms based on deep learning. J Phycol. 2023; doi:10.1111/jpy.13390.
69. Spaulding SA, Potapova MG, Bishop IW, Lee SS, Gasperak TS, Jovanovska E, et al. Diatoms.org: supporting taxonomists, connecting communities. Diatom Research. 2021;36 4:291-304. doi:10.1080/0269249X.2021.2006790.

70. Kloster M, Beszteri B and Nattkemper TW. Annotated Southern Ocean diatom LM micrographs from Polarstern cruises PS79 & PS103. PANGAEA. 2017; doi:10.1594/PANGAEA.914544.
71. Gündüz H, Solak CN and Günal S. Segmentation of diatoms using edge detection and deep learning. Turkish Journal of Electrical Engineering and Computer Sciences. 2022;30 6:2268-85. doi:10.55730/1300-0632.3938.
72. Gündüz H, Solak C and Günal S. Image data set for "Segmentation of diatoms using edge detection and deep learning". 2022; doi:10.34740/kaggle/ds/1187591.
73. Burfeid-Castellanos A, Martín-Martín R, Kloster M, Angulo-Preckler C, Avila C and Beszteri B. Data set accompaying "Epiphytic diatom community structure and richness is determined by macroalgal host and location in the South Shetland Islands (Antarctica)". 2020; doi:10.1594/PANGAEA.925913.
74. Burfeid-Castellanos AM, Martín-Martín RP, Kloster M, Angulo-Preckler C, Avila C and Beszteri B. Epiphytic diatom community structure and richness is determined by macroalgal host and location in the South Shetland Islands (Antarctica). Plos One. 2021;16 4:e0250629.
75. Burfeid-Castellanos AM, Kloster M, Beszteri S, Postel U, Spyra M, Zurowietz M, et al. Data set accompanying "A digital light microscopic method for diatom surveys using embedded acid-cleaned samples". 2022; doi:10.5281/zenodo.5517381.
76. Eerola T, Batrakhanov D, Barazandeh NV, Kraft K, Haraguchi L, Lensu L, et al. Survey of automatic plankton image recognition: challenges, existing solutions and future perspectives. Artificial Intelligence Review. 2024;57 5:114.
77. CEN. UNE-EN 14407: Water quality - Guidance standard for the identification, enumeration and interpretation of benthic diatom samples from running waters. 2014;14407.
78. CEN. UNE-EN 13946:2014 Water quality - Guidance for the routine sampling and preparation of benthic diatoms from rivers and lakes. 2014;13946.
79. Taylor J, Harding W and Archibald C. A methods manual for the collection, preparation and analysis of diatom samples. WRC Report TT 281/07. 2007;Version 1:60.
80. HeliconSoft. Helicon Focus And Focus Stacking. https://wwwheliconsoftcom/heliconsoft-products/helicon-focus/.
81. Chalfoun J, Majurski M, Blattner T, Bhadriraju K, Keyrouz W, Bajcsy P, et al. MIST: accurate and scalable microscopy image stitching tool with stage modeling and error minimization. Scientific reports. 2017;7 1:4988.
82. Preibisch S. ImageJ Grid/Collection Stitching Plugin. https://imagejnet/Grid/Collection_Stitching_Plugin. 2020.
83. Langenkämper D, Zurowietz M, Schoening T and Nattkemper TW. BIIGLE 2.0 - Browsing and Annotating Large Marine Image Collections. Frontiers in Marine Science. 2017;4:83. doi:10.3389/fmars.2017.00083.
84. Trobajo R, Rovira L, Ector L, Wetzel CE, Kelly M and Mann DG. Morphology and identity of some ecologically important small *Nitzschia* species. Diatom research. 2013;28 1:37-59. doi:10.1080/0269249X.2012.734531.
85. Lange-Bertalot H, Hofmann G, Werum M, Cantonati M and Kelly M. Freshwater benthic diatoms of Central Europe: over 800 common species used in ecological assessment. Koeltz Botanical Books Schmitten-Oberreifenberg; 2017.

86. Dosovitskiy A, Beyer L, Kolesnikov A, Weissenborn D, Zhai X, Unterthiner T, et al. An image is worth 16x16 words: Transformers for image recognition at scale. arXiv:201011929. 2020; doi:10.48550/arXiv.2010.11929.
87. Van der Maaten L and Hinton G. Visualizing data using t-SNE. Journal of machine learning research. 2008;9 11.
88. Guo C, Pleiss G, Sun Y and Weinberger KQ. On calibration of modern neural networks. International conference on machine learning. 2017:1321-30.
89. Abdar M, Pourpanah F, Hussain S, Rezazadegan D, Liu L, Ghavamzadeh M, et al. A review of uncertainty quantification in deep learning: Techniques, applications and challenges. Information fusion. 2021;76:243-97.
90. Venkataramanan A, Benbihi A, Laviale M and Pradalier C. Gaussian Latent Representations for Uncertainty Estimation using Mahalanobis Distance in Deep Classifiers. Proceedings of the IEEE/CVF International Conference on Computer Vision. 2023:4488-97.
91. Pelleg D. Extending K-means with efficient estimation of the number of clusters in ICML. Proceedings of the 17th international conference on machine learning. 2000:277-81.
92. Schroff F, Kalenichenko D and Philbin J. Facenet: A unified embedding for face recognition and clustering. In: *Proceedings of the IEEE conference on computer vision and pattern recognition* 2015, pp.815-23.
93. Liu J, Lin Z, Padhy S, Tran D, Bedrax Weiss T and Lakshminarayanan B. Simple and principled uncertainty estimation with deterministic deep learning via distance awareness. Advances in neural information processing systems. 2020;33:7498-512.
94. Van Amersfoort J, Smith L, Teh YW and Gal Y. Uncertainty estimation using a single deep deterministic neural network. In: *International conference on machine learning* 2020, pp.9690-700. PMLR.
95. Li J, Chen P, He Z, Yu S, Liu S and Jia J. Rethinking out-of-distribution (ood) detection: Masked image modeling is all you need. In: *Proceedings of the IEEE/CVF conference on computer vision and pattern recognition* 2023, pp.11578-89.
96. He K, Chen X, Xie S, Li Y, Dollár P and Girshick R. Masked autoencoders are scalable vision learners. Proceedings of the IEEE/CVF conference on computer vision and pattern recognition. 2022:16000-9.
97. Chen T, Kornblith S, Norouzi M and Hinton G. A simple framework for contrastive learning of visual representations. International conference on machine learning. 2020:1597-607.
98. He K, Fan H, Wu Y, Xie S and Girshick R. Momentum contrast for unsupervised visual representation learning. Proceedings of the IEEE/CVF conference on computer vision and pattern recognition. 2020:9729-38.
99. Xie Z, Zhang Z, Cao Y, Lin Y, Bao J, Yao Z, et al. Simmim: A simple framework for masked image modeling. Proceedings of the IEEE/CVF Conference on Computer Vision and Pattern Recognition. 2022:9653-63.
100. Bao H, Dong L, Piao S and Wei F. Beit: Bert pre-training of image transformers. arXiv:210608254. 2021; doi:10.48550/arXiv.2106.08254.
101. Zhou J, Wei C, Wang H, Shen W, Xie C, Yuille A, et al. ibot: Image bert pre-training with online tokenizer. arXiv:211107832. 2021; doi:10.48550/arXiv.2111.07832.
102. Sokolova M and Lapalme G. A systematic analysis of performance measures for classification tasks. Information processing & management. 2009;45 4:427-37.

103. Venkataramanan A, Kloster M, Burfeid-Castellanos A, Dani M, Mayombo NAS, Vidakovic D, Langenkämper D, Tan M, Pradalier C, Nattkemper T, Laviale M, Beszteri B (2024): Supporting data for "“UDE DIATOMS in the Wild 2024”: A new image dataset of freshwater diatoms for training deep learning models" GigaScience Database. https://doi.org/10.5524/102580.
104. Kloster M, Burfeid-Castellanos A, Dani M, Mayombo N A S, Beszteri B, Vidaković D. UDE Diatoms in the Wild 2024. Zenodo. 2024 https://doi.org/10.5281/zenodo.10410655
105. Kloster M, Burfeid-Castellanos A, Dani M, Mayombo N A S, Vidaković D, Beszteri B. UDE Diatoms in the Wild 2024 - subsets of taxa. Kaggle repository. 2024 https://www.kaggle.com/datasets/michaelkloster/ude-diatoms-in-the-wild-2024

Figure legends

Figure 1. Selected examples of diatom specimens. Valvar views from three different genera (Navicula, Encynoema, Planothidium), each one with visually highly similar but distinct species.

Figure 2: Illustrations of some challenges of visual diatom identification. a) Due to the complex live cycle, the frustule size reduction usually leads to a change in length-to-width ratio, resulting in different visual appearance. b) Diatoms can also present ecomorphological variability, i.e. a species can vary in form depending on environmental influences. c) Diatoms can also vary their morphological traits such as valve ornamentation within a single species (phenotypic plasticity / morphological variability). d) Valve orientation relative to the imaging optical axis gives different visual appearances: valvar vs. pleural views refer to viewing angles roughly perpendicular to each other and occur most commonly, depending on the species. Intermediate (oblique or tilted) perspectives can usually be found much less frequently. e) Large diatom species complexes (sensu lato taxon groups) can add to morphological variability. One of many examples is Cocconeis placentula sensu lato, which includes Cocconeis placentula, Cocconeis euglypta, Cocconeis lineata and Cocconeis pseudolineata. f) Monoraphid diatoms possess two valves with different morphological appearances, where only one valve presents a raphe (i.e. an elongated slit), the other not (raphe and rapheless valves, respectively).

Figure 3: Example of a "real life" diatom preparation. These can, as in this case, contain complex a background (sediment particles and diatom fragments) as well as diatom valves overlapping with each other.

Figure 4. The ten most abundant species visualized in a scatter plot using t-SNE dimensionality reduction. Colors indicate species membership. Each data point depicts one cutout.

Figure 5. Abundance distribution of the 144 classes with at least 50 examples, illustrating the data imbalance typical of biodiversity datasets.

Figure 6. Pipeline for OOD sample detection in diatoms using MAPLE (Experiment 1). During training, heterogeneous classes are split into subclasses by X-means clustering, resulting in refined labels (corresponding to these subclasses / clusters). A triplet loss supports separation of classes. During inference, a PCA projection learned during the training phase is applied to feature embeddings and is used as input for a Mahalanobis-distance-based uncertainty quantification and OOD sample detection.

Figure 7. Receiver operating characteristic curves from the OOD sample detection experiment (Experiment 1) for the Deterministic vs. MAPLE methods on the D50 and on the D25 datasets.

Figure 8. Examples illustrating subclusters within individual species delimited by MAPLE. a-b) Achnanthidium atomoides in pleural (a) vs. valvar view (b); c-d) Amphora pediculus, represented as single valve (c) vs. both valves together (d); e-f) subclusters in Fragilaria pectinalis appear to depict life cycle associated variants.

Figure 9. Structure of datasets for Experiment 2. 20% of the images were used as test set (D^{test}). In one experiment, all the remaining (80%) images were used for model training (denoted D^t on the left-hand side). In a second experiment, only 10% of training data of each class in D^t (denoted $D^t_{0.1}$ on the right-hand side) was used for model training to investigate the effect of dataset size.

Figure 10. Fowchart of Experiment 2. Pre-training refers to a supervised training for the baseline model (ViT), and a pretext training for the semi-supervised model (MAE). Finally, all models were fine-tuned in a supervised fashion.

Tables

Table 1 Overview of existing diatom image datasets.

Dataset/ Project name	Authors	# of images	# of species	Link to the dataset
ADIAC	Du Buf et al. 2000 [24, 62]	3,400	328	https://websites.rbge.org.uk/ADIAC/db/adiacdb.htm
Aqualitas	Bueno et al. 2020 [45, 63, 64]	10,000	100	https://figshare.com/articles/dataset/Aqualitas_Database_full_release_/11728980
Synthetic dataset for diatom automatic detection	Laviale et al. 2023 [35]	9,230	166	https://dorel.univ-lorraine.fr/dataset.xhtml?persistentId=doi:10.12763/UADENQ
Southern Ocean diatoms (PS79/PS103)	Kloster et al. 2017 [46, 70]	3,300	10	https://doi.pangaea.de/10.1594/PANGAEA.914544
Kaggle, Diatom Dataset	Gündüz et al. 2022 [71, 72]	3,027	68	https://www.doi.org/10.34740/kaggle/ds/1187591
Antarctic Epiphytes	Burfeid-Castellanos	18,441	120	https://doi.pangaea.de/10.1594/PANGAEA.925913

	et al. 2021 [73, 74]			
UDE PhycoLab Menne	Burfeid-Castellanos et al. 2022 [18, 75]	8,858	161	https://zenodo.org/record/5517381
Kaggle, scraped from Diatoms.org	Pu et al. 2023 [68]	7,983	1,042	https://www.kaggle.com/datasets/siyuepu/diatom-datasets
UDE DIATOMS in the Wild 2024	This paper	83,570	611	https://doi.org/10.5281/zenodo.10410655, https://www.kaggle.com/datasets/michaelkloster/ude-diatoms-in-the-wild-2024

Table 2. Metadata files of the dataset

Column	Content
annotation_id	original BIIGLE annotation id (unique ID within the dataset)
type	type of diatom morphology according to the "Diatoms of North America" identification key (https://diatoms.org/morphology)
genus	genus of the annotated specimen
species	species of the annotated specimen ("None" if not identified to species level)
subspecies	historical subspecies or species complex of the annotated specimen, might be shifted to a different species in the near future ("None" if not identified to subspecies level)
annotator	id of the annotator
bbox_x0, bbox_y0, bbox_x1, bbox_y1	coordinates of the cutout within the original virtual slide image (axis-parallel bounding box, with roughly manually defined borders)
shape	type of annotation shape (“Polygon”, “Circle” or “Rectangle”)
points	coordinates of the points of the annotation shape. For Polygon = [x0, y0, y1, y1, ...], for Circle = [x, y, r], for Rectangle = [x0, y0, x1, y1, x2, y2, x3, y3] (rotated bounding box)
image_id	the original BIIGLE image id
image_filename	the filename of the virtual slide image the annotation was cut out
cutout_filename	the filename of the cutout

Table 3. Evaluation metrics for the OOD sample detection experiment (Experiment 1). For a given dataset, a metric score in bold is higher when comparing deterministic and MAPLE methods.

Dataset	Method	Accuracy	F1-score	AUROC	AUPR
D25	Deterministic	**72.60 %**	0.5622	0.8046	0.8243
D25	MAPLE	71.75 %	0.5610	**0.8388**	**0.8421**
D50	Deterministic	60.41 %	0.5639	0.6844	0.6618
D50	MAPLE	**76.65 %**	0.5531	**0.7282**	**0.7145**

Table 4. Evaluation metrics for Experiment 2. A metric score in bold is higher when comparing ResNet50 (referred to as RN), ViT and MAE methods.

Experiment	Macro-average accuracy	Micro-average accuracy	Macro-average F1-score	Macro-average AUROC score
RN_{D^t}	63.76%	78.78%	0.6507	0.9798
ViT_{D^t}	60.31%	78.04 %	0.6283	0.9490
MAE_{D^t}	**66.37 %**	**80.61 %**	**0.6824**	**0.9848**
$RN_{D^t_{0.1}}$	41.78%	70.04%	0.4315	0.9421
$ViT_{D^t_{0.1}}$	42.19 %	69.97 %	0.4456	0.9397
$MAE_{D^t_{0.1}}$	**47.75 %**	**73.22 %**	**0.4941**	**0.9821**

 Click here to access/download;Figure;Figure 1.png

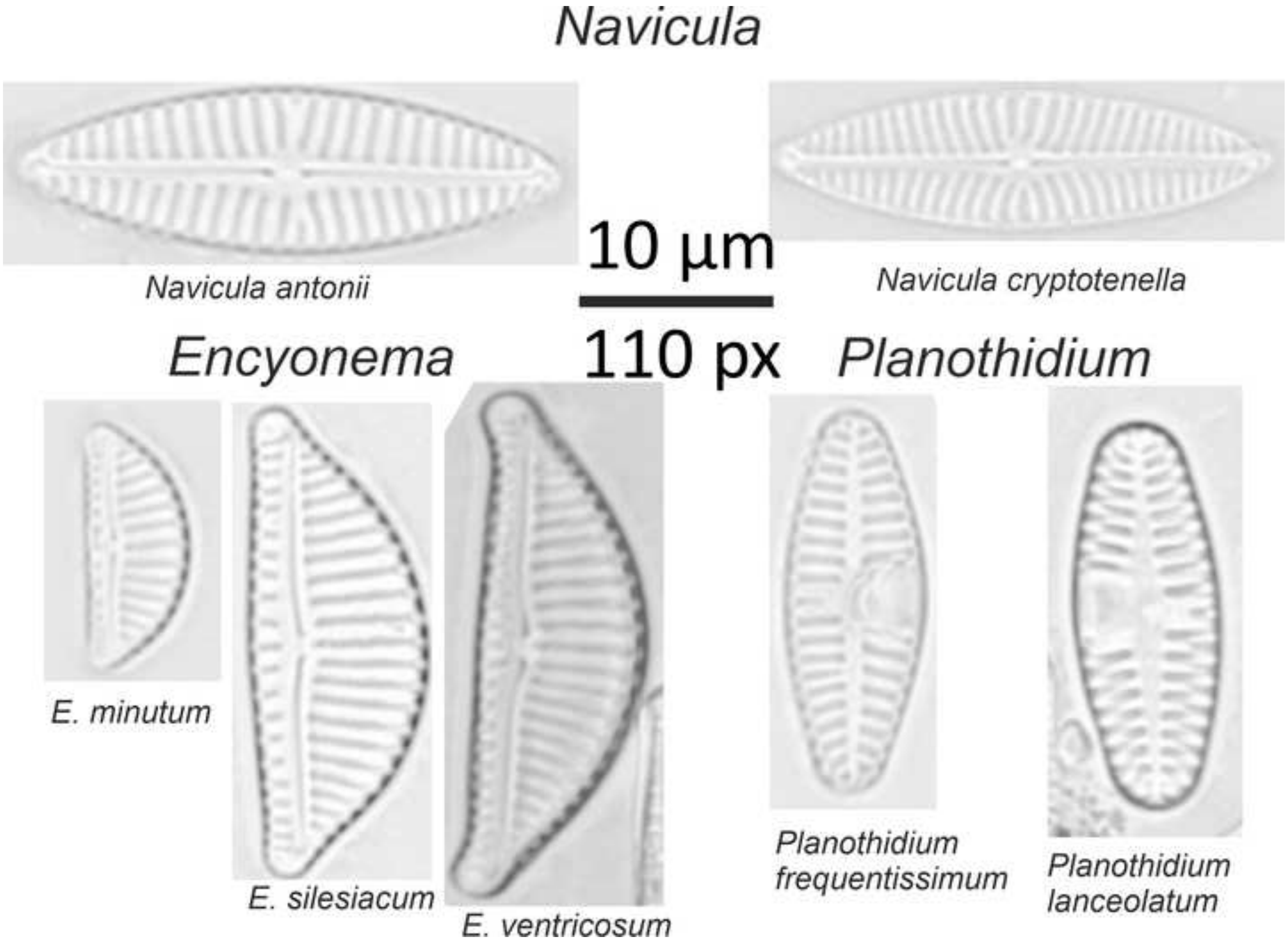

Figure
Click here to access/download;Figure;Figure 2.png

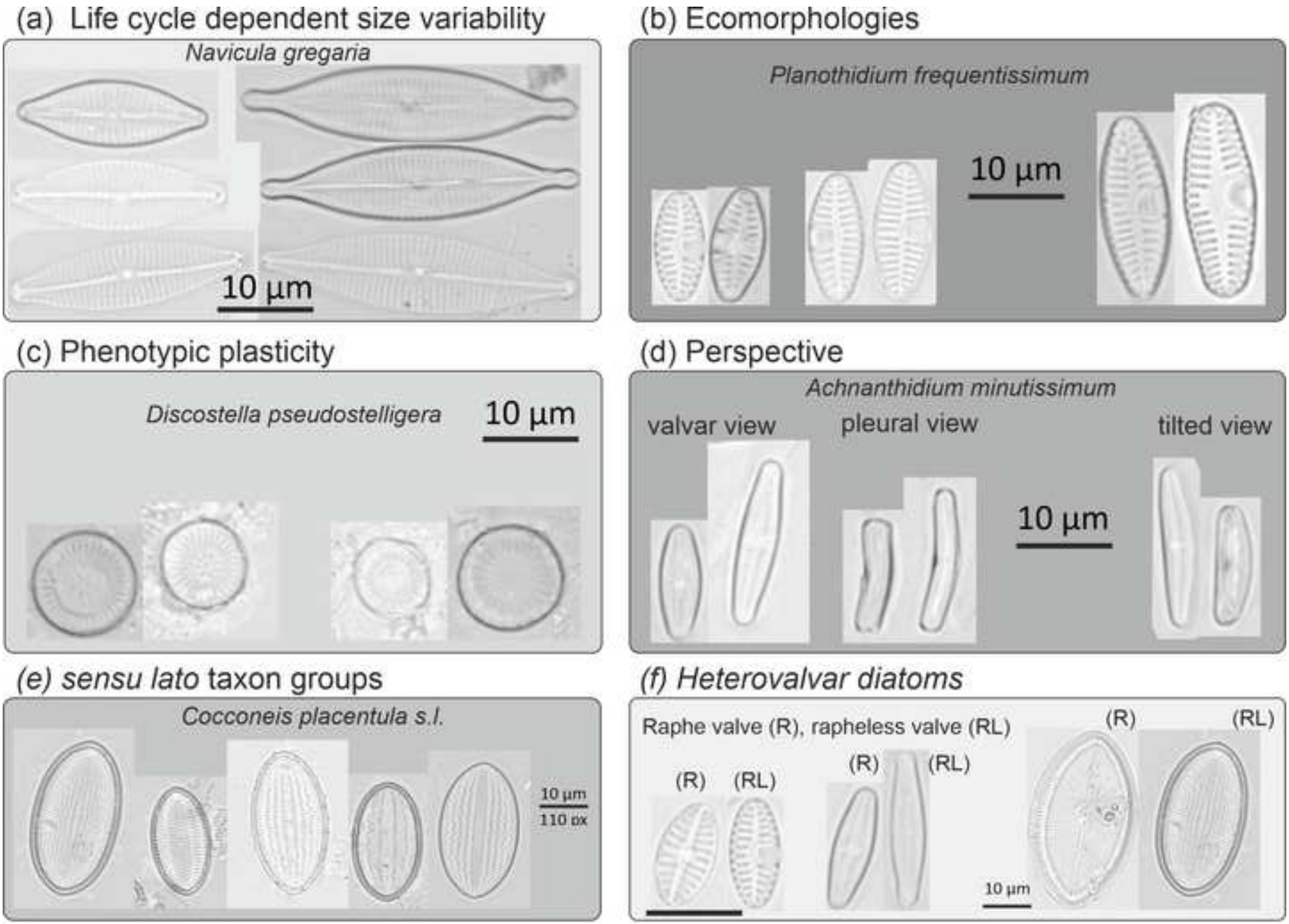

Figure Click here to access/download;Figure;Figure 3.png

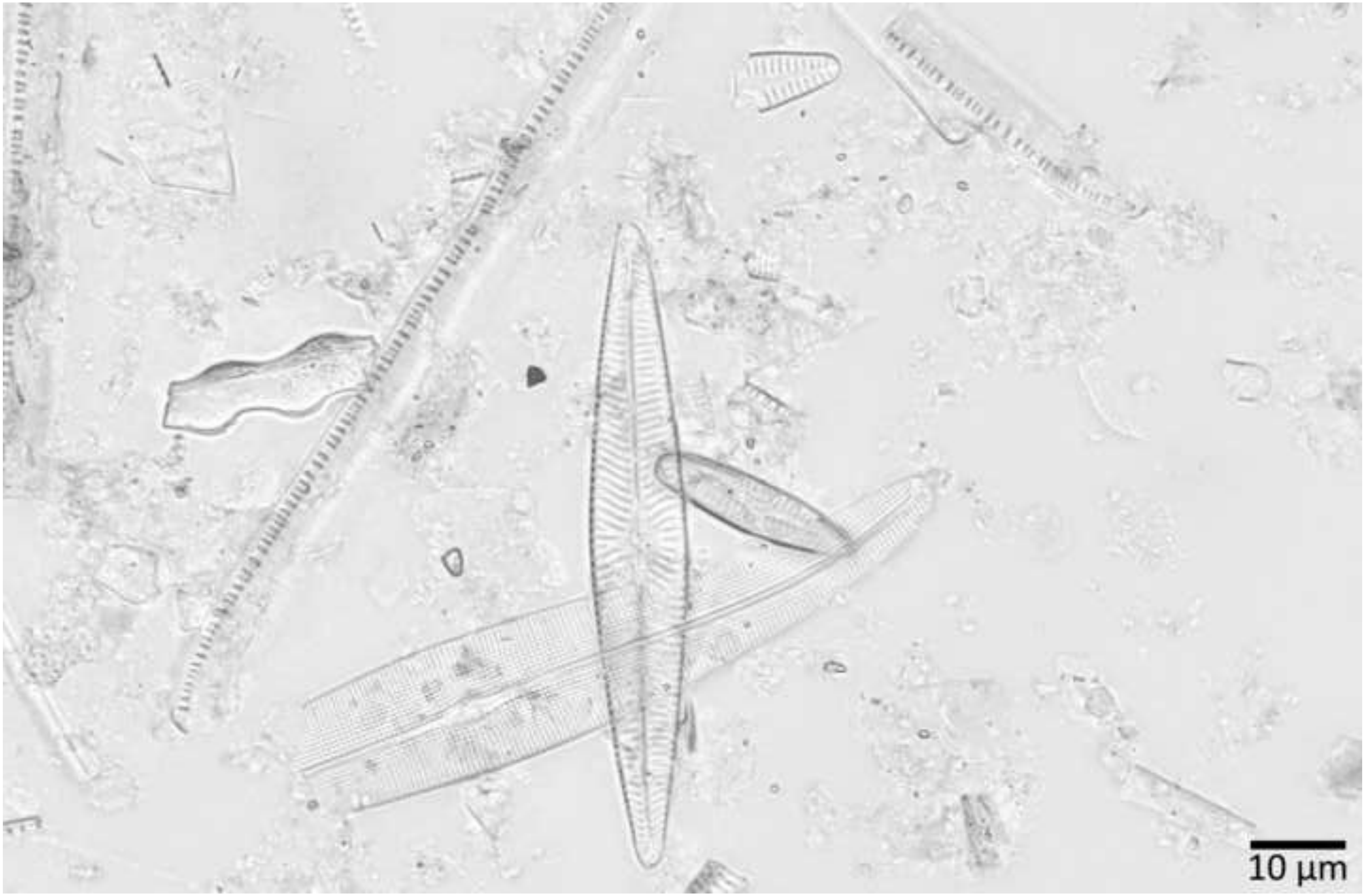

Figure

Click here to access/download;Figure;Figure 4.png

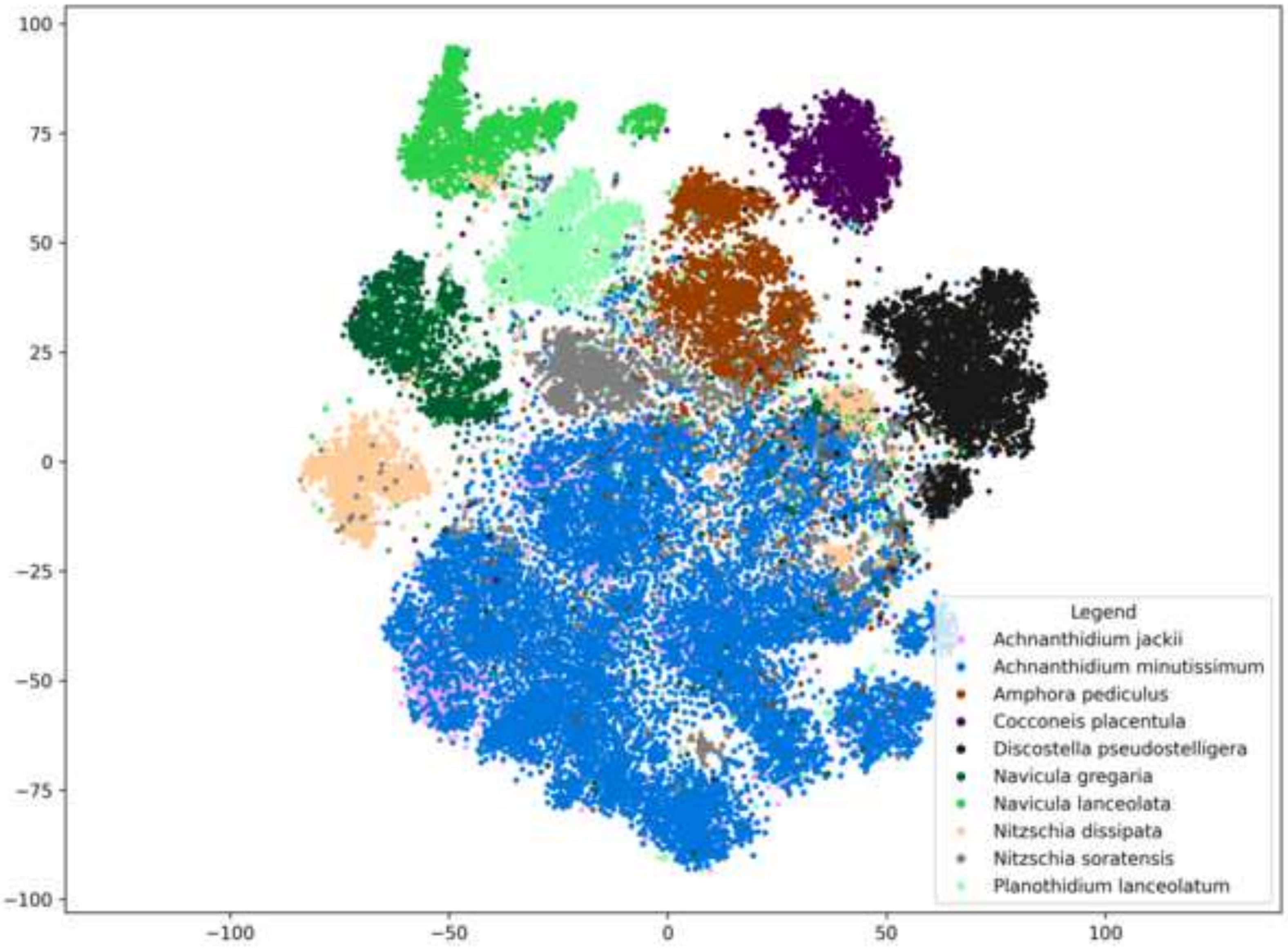

 Click here to access/download;Figure;Figure 5.png

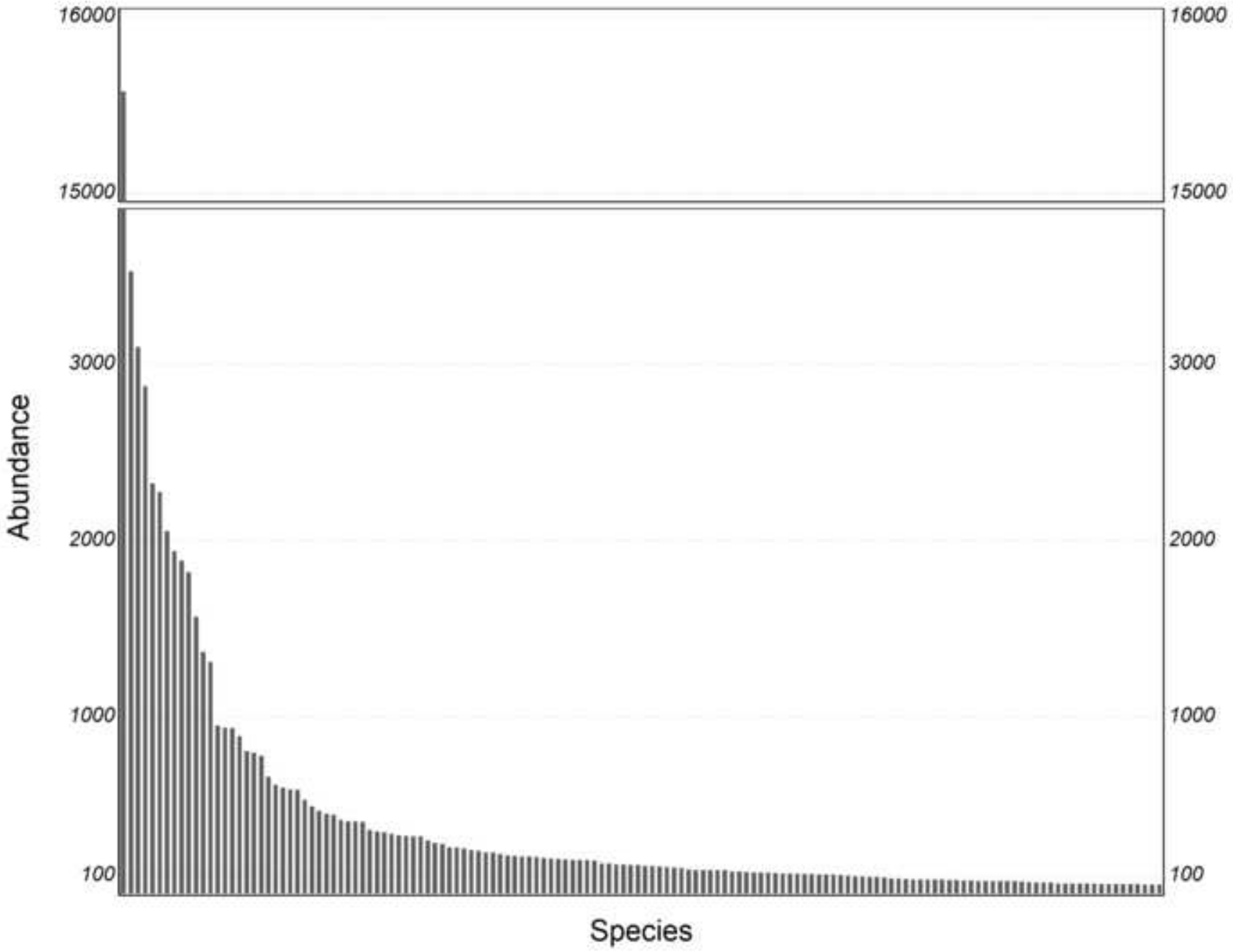

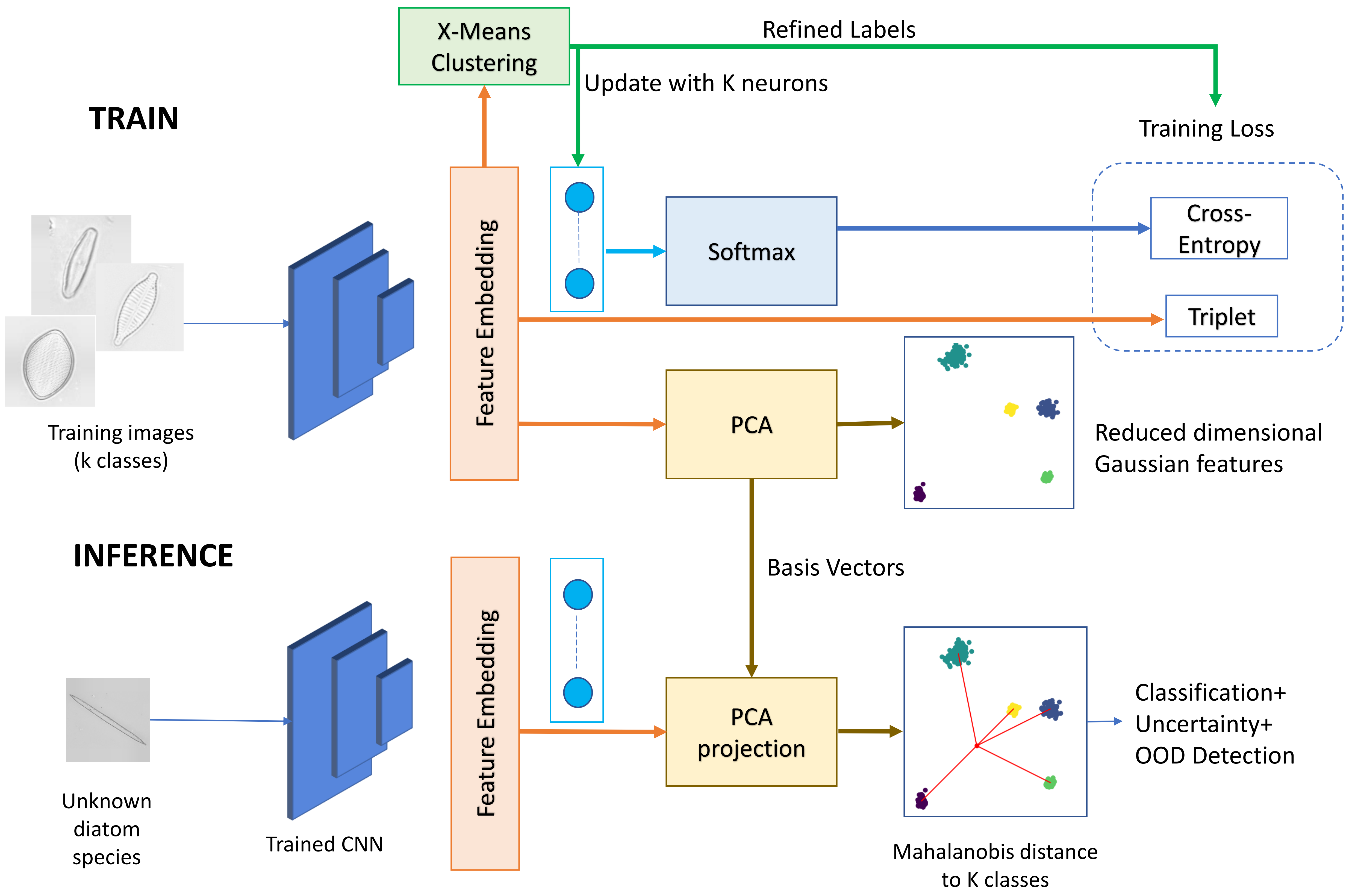
X-Means Clustering
Refined Labels
Update with K neurons
TRAIN
Training Loss
Softmax
Cross-Entropy
Triplet
Feature Embedding
Training images (k classes)
PCA
Reduced dimensional Gaussian features
INFERENCE
Basis Vectors
Feature Embedding
PCA projection
Classification+ Uncertainty+ OOD Detection
Unknown diatom species
Trained CNN
Mahalanobis distance to K classes

Figure Click here to access/download;Figure;Figure 7.png

(a)

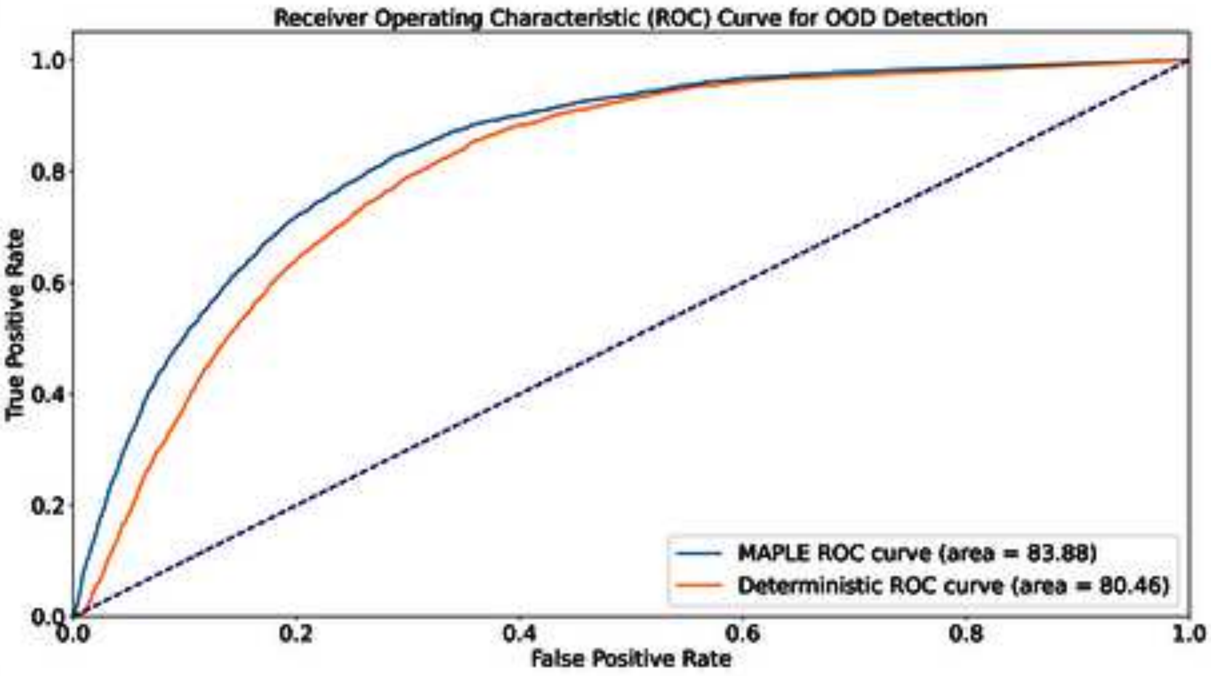

(b)

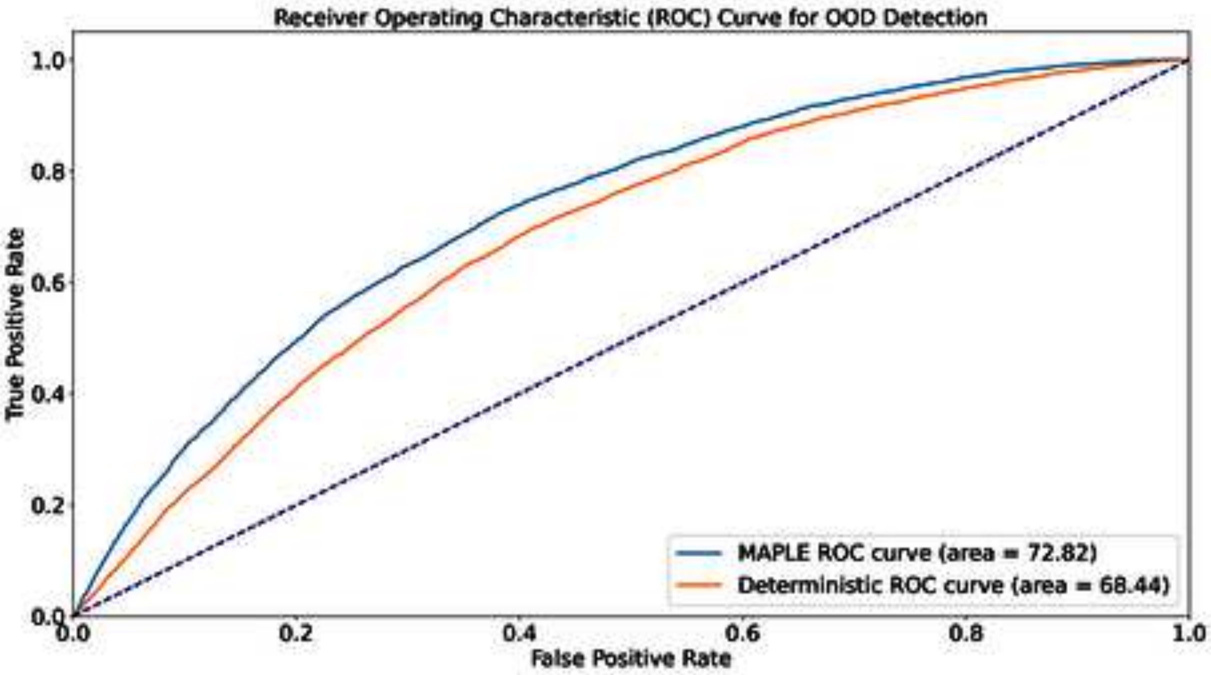

Figure

Click here to access/download;Figure;Figure 8.pdf

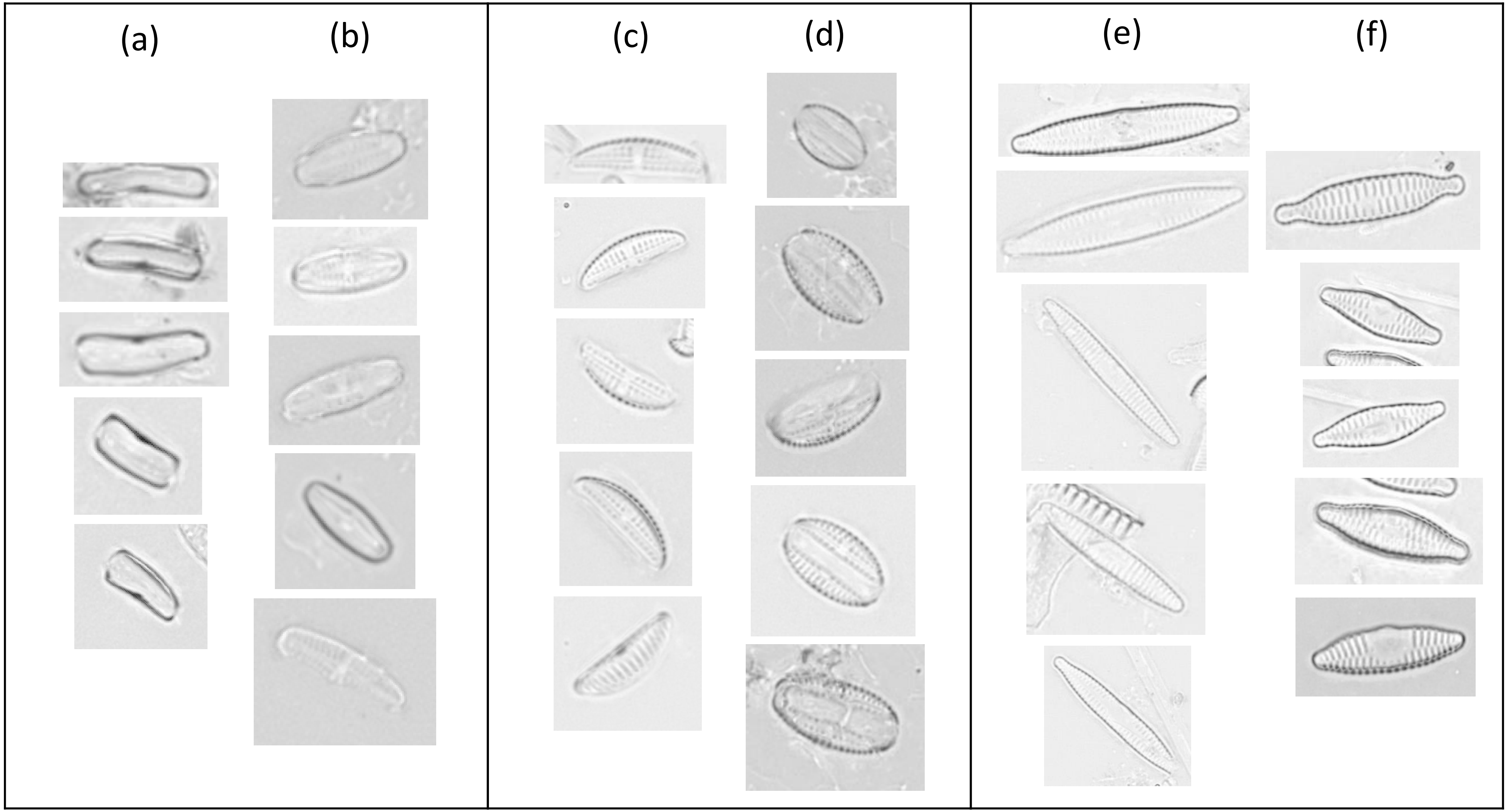

 Click here to access/download;Figure;Figure 9.png

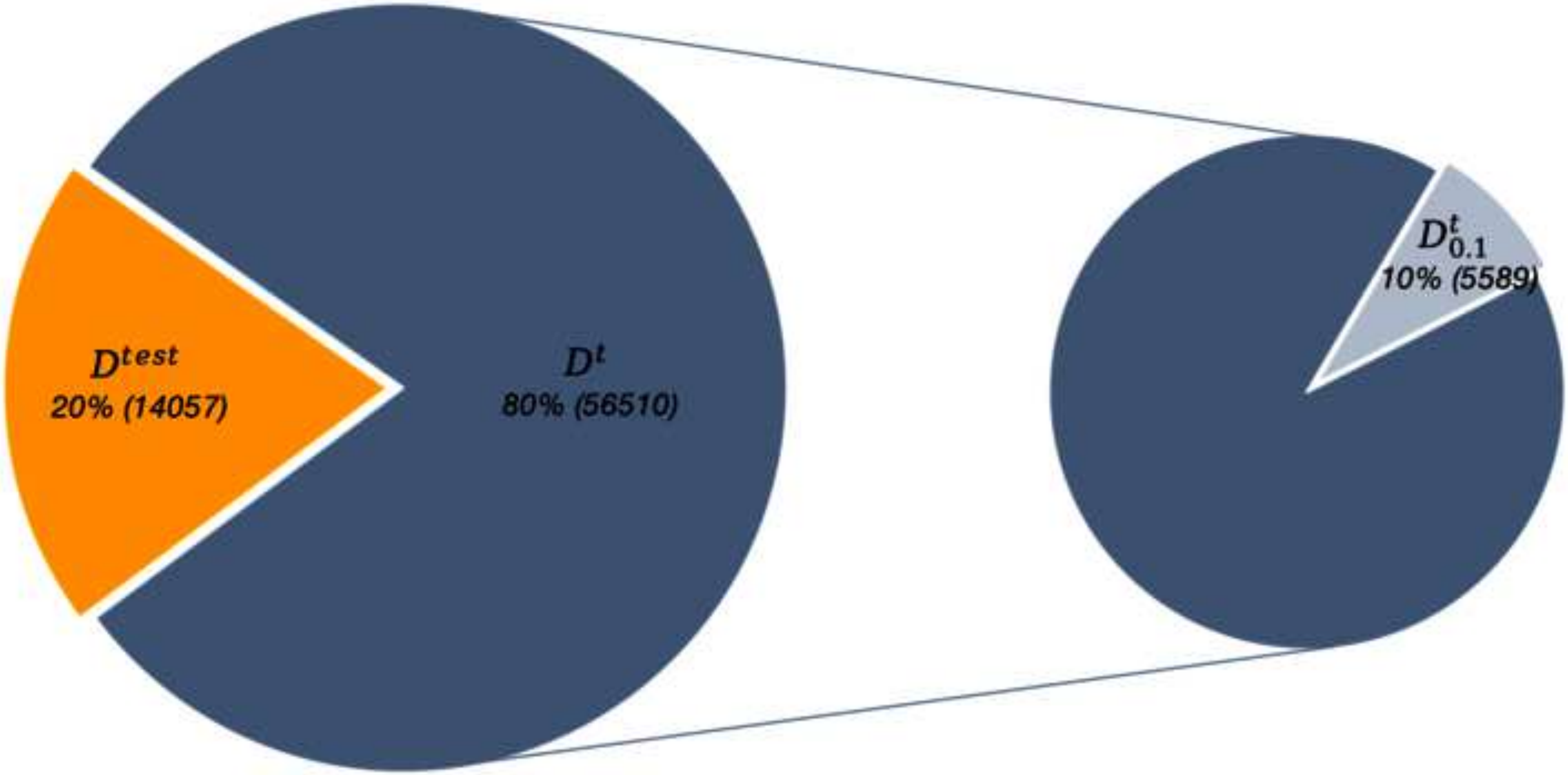

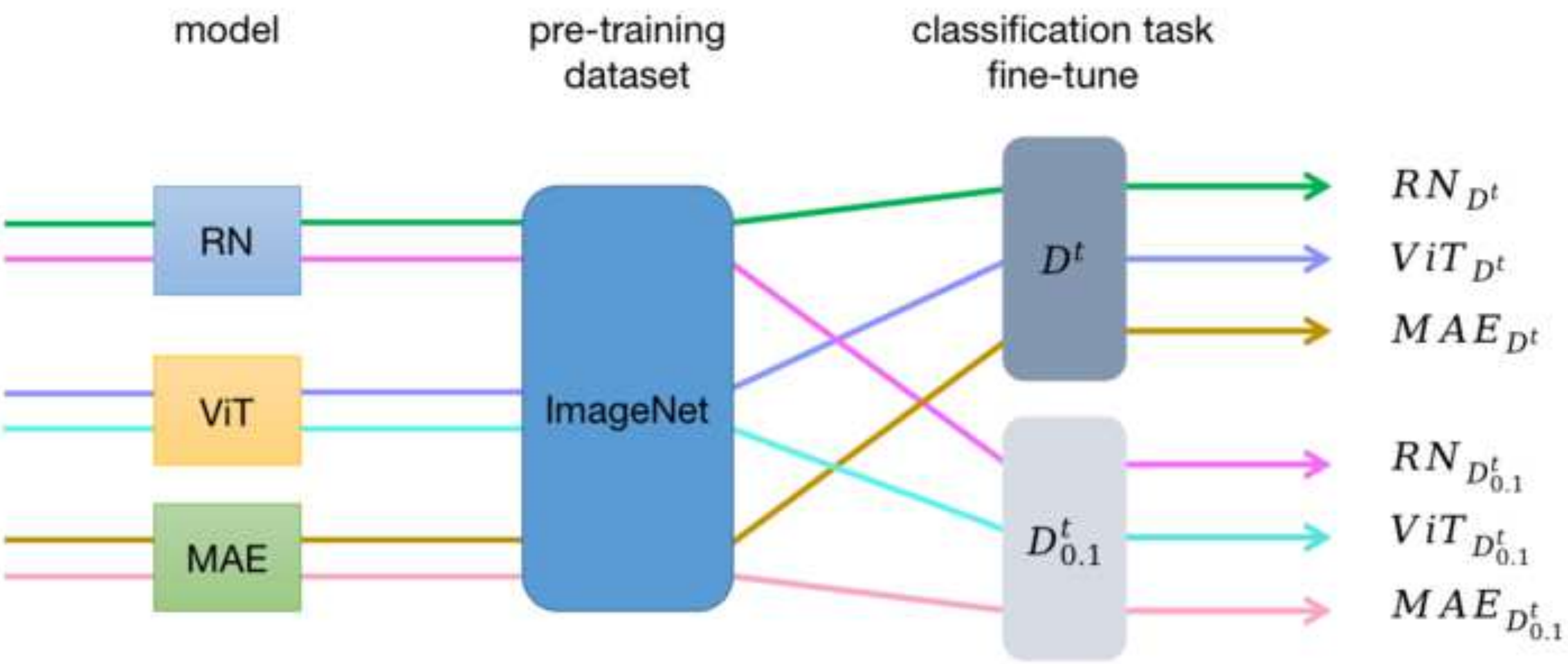
model
pre-training dataset
classification task fine-tune
RN
ViT
MAE
ImageNet
D^t
$D^t_{0.1}$
RN_{D^t}
ViT_{D^t}
MAE_{D^t}
$RN_{D^t_{0.1}}$
$ViT_{D^t_{0.1}}$
$MAE_{D^t_{0.1}}$

Click here to access/download
Supplementary Material
Supplement Figure 1 - tSNE.html

Click here to access/download
Supplementary Material
Supplement Script 1 - Convert to DatasetFolder structure.R

Click here to access/download
Supplementary Material
Supplement Table 1 - Metadata Samples and Slides.xlsx

Click here to access/download
Supplementary Material
Supplement Table 2 - Species abundance.docx
