# Supplementary material for: “UDE DIATOMS in the Wild 2024”: a new image dataset of freshwater diatoms for training deep learning models
Source: Gigascience. 2024 Nov 28;13:giae087. doi: 10.1093/gigascience/giae087 (PMC11604061; doi:10.1093/gigascience/giae087)
Supplement: giae087_Response_to_Reviewer_Comments_Revision_1 [file giae087_response_to_reviewer_comments_revision_1.pdf]

Author's Response To Reviewer Comments

Dear Hans,

I uploaded the manuscript file after implementing the requested changes.
As to your question about the redundance between the Zenodo and Kaggle repositories: the co-authors would like to have the data on Kaggle as a "living" version of the data set that can be updated in the future. That is the reason for not freezing it with a DOI (I previously dicussed this point with the database editor as well). So I kept both the Zenodo DOI, as well as the Kaggle URL. I hope this is ok also from your side.

Best regards,
Bank
