# Supplementary material for: “UDE DIATOMS in the Wild 2024”: a new image dataset of freshwater diatoms for training deep learning models
Source: Gigascience. 2024 Nov 28;13:giae087. doi: 10.1093/gigascience/giae087 (PMC11604061; doi:10.1093/gigascience/giae087)
Supplement: giae087_Response_to_Reviewer_Comments_Revision_2 [file giae087_response_to_reviewer_comments_revision_2.pdf]

Author's Response To Reviewer Comments

Dear Hans,

done as requested, the links from Table 1 were moved into the list of citations. Wherever a doi was available, it was used. Also, we have updated the license for our dataset on Kaggle. As to the doi for the latter, as discussed previously, we did not want to freeze this version of the dataset. It is available as used in the manuscript via Zenodo; the Kaggle version is meant to be updated in the future. You (as well as a dataset editor) have agreed to this treatment previously.

I hope we now addressed all open points.

Best wishes,
Bank
