# Supplementary material for: “UDE DIATOMS in the Wild 2024”: a new image dataset of freshwater diatoms for training deep learning models
Source: Gigascience. 2024 Nov 28;13:giae087. doi: 10.1093/gigascience/giae087 (PMC11604061; doi:10.1093/gigascience/giae087)
Supplement: giae087_Reviewer_1_Report_Original_Submission — Sarah Spaulding -- 6/19/2024 [file giae087_reviewer_1_report_original_submission.pdf]

Reviewer Report

Title: â€œUDE DIATOMS in the Wild 2024â€: A new image dataset of freshwater diatoms for training deep learning models

Version: Original Submission **Date:** 6/19/2024

Reviewer name: Sarah Spaulding

Reviewer Comments to Author:

"UDE DIATOMS in the Wild 2024": A new image dataset of freshwater diatoms for training deep learning modelsGeneral Comments:The rationale provided for the purpose of the dataset is to improve the objective, reproducible and comparable nature of diatom data. The components of the problem seems to be 1) obtaining images, and what is done to them - annotation and resolving differing annotations; and 2) evaluating what deep learning seems to produce from the images. Notably, the images are not idealized, but have all of the complications of specimens encounter by human microscopists.The abstract uses "findings" which does not seem to fit the context. Similarly, "conclusions" could be stating the informative outcomes of the two experiments.I am not a specialist in deep learning, but a diatom biologist. Therefore, I am able to comment on the more non-technical aspects of the manuscript. In particular, I note words and phrases that could improve clarity of the text. The authors might consider providing additional use cases for those interested in obtaining the data.The manuscript is appropriate for publication in Gigascience, pending minor revisions.Specific Comments:Line 24. "need for digitalisation of these methods has long been recognized". I was not aware of the term digitalization and its meaning. Consider adding the definition. Wording would be is vague - is it really the methods that are digitalized? Or by "methods", do you mean to include image capture and AI recognition?Line 25. One might argue that diatoms lack morphological diversity. Compared to other organisms, diatoms tend to be identified by subtle shape features; they are lacking in the number of morphological features they possess.Line 31. As a dataset, "findings" is not an outcome of the product. Consider "data descriptor" or alternate term.Line 39. Similarly, the text here does not represent "conclusions", but a restatement of the problem.Line 62. Omit "these" in "A need for digitalisation of light microscopic methods". So you include "identification" in the method?Line 68. "performed directly on a microscope" - Do you mean "performed by microscopists" or "performed by diatomists, using a microscope"?Line 70. Consider rewording as, "Over 20 years ago, the ADIAC project developed fundamental approaches for digital imaging and identification [24]. More than ever, we now need standardized, digital imaging methods combined with automated (?) taxonomic identification in order to have objective, reproducible, and comparable taxonomic data for rapid processing of large numbers of samples".Line 84. Suggested edit to, "solved by focusing up and down through the three-dimensional structure of a valve, termed 'optical dissection' ".Line 89. Note that some software applications (at least Olympus software) are able to compare the degree of blur in a stack and save the stratum with the sharpest focus.Line 100. What is the difference between a gigapixel-sized slide scan or a virtual slides? Clarify, as these seem like the same thing to me.Line 103. Replace, "There are a number of factors" with "Several factors"Line 105. Edit to "local or regional"Line 111. Omit "highly", as something is either time-consuming or not.Line 134. Omit

"highly"Line 136. Consider edit to, "Human analysts learn to interpret and link differing orientations with experience"Line 159. Consider edit to, "Thus, analyses of light microscopic images of diatoms by deep learning is an urgent need for research of ecology and biodiversity, as well as environmental monitoring. Yet, development of the machine learning and computer vision is a challenge.Line 163. What is "benchmarking"? Not being a specialist, I need clarification. OR could this sentence be just as meaningful by stating, "datasets that are suitable for training deep learning models". What is the importance of benchmarking?Line 163. "There are very few extensive taxonomically annotated diatom image datasets publicly available to begin with, and the available ones are mostly too small to be suitable for training deep learning models." This would be more clearly written as,"Few diatom image datasets have associated taxonomic annotations, and of those that do have annotations, the datasets are generally too small to be suitable for training deep learning models."Line 167. "pre-deep learning machine" is sort of a weird phrase.Line 177. Cite diatoms.org as:Spaulding, S.A., Potapova, M.G., Bishop, I.W., Lee, S.S., Gasperak, T.S., Jovanovska, E., and Edlund, M.B. 2021. Diatoms.org: supporting taxonomists, connecting communities. Diatom Research 36(4): 291-304. doi:10.1080/0269249X.2021.2006790Line 180. Cite Kaggle as:https://www.kaggle.com/ - unless they have an alternate citation.Line 197. "is being made publicly available to support customizing and benchmarking deep learning models to this field of application."Is it available now? Can you state, "is publicly available to support customizing and benchmarking deep learning models for diatoms".Line 290. I would think the header should be "Potential uses"Line 291. "Challenging nature of the dataset" sounds like the dataset is difficult to use, rather than (and I think this is what you mean), "deep learning as applied to diatoms is challenging because…." Clarify, especially because it seems that "challenging" is defined diffently in the abstract. Suggested revision to place the purpose of each experiment first:We present two deep learning experiments, each addressing a particular challenge of deep learning as applied to diatom analysis. The first experiment is useful for illustrating the situation in which specimens of taxa are encountered that were not present in the training set. We expect the experiment to demonstrate (?) the distinct visual appearance of valves lying in orientations not previously encountered (?). The first experiment uses a deep learning approach to handle the detection of out-of-distribution samples and explicitly models intra-class heterogeneity.The second experiment is useful for investigating the potential of semi-supervisedlearning (SSL) to alleviate the need for human expertise to annotate image collections.Here, SSL simulates unlabelled image data to learn better feature representations. The results are compared to a study conducted with a vision transformer model.Line 333. What is "triplet loss"?Line 389. What is "DL"?Line 390. Would be better written as "Our results show x process produced lower accuracies of y,z"Line 394. Okay, now I understand why you say the dataset is challenging. But this differs from earlier definition - you can also add that the dataset is more reflective of the typical use case.Line 407. What is "presence of occlusions within the dataset"?Line 411.Who is "computer vision community". If this is your audience, make that clear early on rather than at the end.

Level of Interest

Please indicate how interesting you found the manuscript: Choose an item.

Quality of Written English

Please indicate the quality of language in the manuscript: Choose an item.

Declaration of Competing Interests

Please complete a declaration of competing interests, considering the following questions:

- Have you in the past five years received reimbursements, fees, funding, or salary from an organisation that may in any way gain or lose financially from the publication of this manuscript, either now or in the future?
- Do you hold any stocks or shares in an organisation that may in any way gain or lose financially from the publication of this manuscript, either now or in the future?
- Do you hold or are you currently applying for any patents relating to the content of the manuscript?
- Have you received reimbursements, fees, funding, or salary from an organization that holds or has applied for patents relating to the content of the manuscript?
- Do you have any other financial competing interests?
- Do you have any non-financial competing interests in relation to this paper?

If you can answer no to all of the above, write 'I declare that I have no competing interests' below. If your reply is yes to any, please give details below.

I declare that I have NO competing interests.

I agree to the open peer review policy of the journal. I understand that my name will be included on my report to the authors and, if the manuscript is accepted for publication, my named report including any attachments I upload will be posted on the website along with the authors' responses. I agree for my report to be made available under an Open Access Creative Commons CC-BY license (http://creativecommons.org/licenses/by/4.0/). I understand that any comments which I do not wish to be included in my named report can be included as confidential comments to the editors, which will not be published.

Choose an item.

To further support our reviewers, we have joined with Publons, where you can gain additional credit to further highlight your hard work (see: https://publons.com/journal/530/gigascience). On publication of this paper, your review will be automatically added to Publons, you can then choose whether or not to claim your Publons credit. I understand this statement.

Yes Choose an item.
