# Supplementary material for: “UDE DIATOMS in the Wild 2024”: a new image dataset of freshwater diatoms for training deep learning models
Source: Gigascience. 2024 Nov 28;13:giae087. doi: 10.1093/gigascience/giae087 (PMC11604061; doi:10.1093/gigascience/giae087)
Supplement: giae087_Reviewer_2_Report_Original_Submission — Tuomas Eerola -- 6/20/2024 [file giae087_reviewer_2_report_original_submission.pdf]

Reviewer Report

Title: â€œUDE DIATOMS in the Wild 2024â€: A new image dataset of freshwater diatoms for training deep learning models

Version: Original Submission **Date:** 6/20/2024

Reviewer name: Tuomas Eerola

Reviewer Comments to Author:

The paper introduces a new image dataset for training and testing models for diatom recognition. The dataset is considerably larger than previous datasets and provides a more realistic benchmark for diatom recognition methods than earlier 'clean' datasets. Due to the fine-grained nature of the diatom recognition task, the dataset has the potential to be useful in deep learning model development beyond the application area. Therefore, the dataset is definitely worth publishing. However, due to the shortcomings in the manuscript, I cannot recommend its publication in its current form.The main comments:1) The set of experiments included in the paper is somewhat unusual for a dataset paper. I would expect to see baseline experiments of common (closed-set) recognition models (ResNet, ViT, etc.) with the entire dataset. Instead, the authors provide two rather specific experiments; one on out-of-distribution detection and one on self-supervised learning. While these are both interesting experiments, it is unclear why they were selected and leave the purpose of the dataset a bit unclear. I would recommend including baseline plankton recognition experiments.2) The paper lacks a clearly defined evaluation protocol. One of the main problems with most existing diatom datasets, and plankton recognition datasets in general, is that there is no standardized way to use them. This leads to a situation where different papers apply the data differently (different splits, evaluation metrics, etc.), and the results in different papers are not comparable despite the use of the same dataset. The paper would greatly benefit from a clear description of the evaluation protocol that everyone using the dataset could follow and replicate the experiments with their method.Detailed comments3) In Table 1, the authors list existing image datasets that consist of only diatoms. While the table appears comprehensive, there is also a large pool of more general phytoplankton datasets that contain various diatom taxa. Some of these could also be listed. For example, see:Eerola, T., et al. (2024). Survey of automatic plankton image recognition: Challenges, existing solutions, and future perspectives. Artificial Intelligence Review, 57(5), 114.4) More details on the annotation process should be provided. How many annotators in total? How many annotators per image? Was label uncertainty/conflicting expert labels addressed in some way? In lines 257-258, the authors state that 'The annotations were ... filtered to remove irrelevant labels and annotations from inexperienced annotators.' What does this mean?5) The term 'out-of-distribution detection' is commonly used but a bit confusing in this context because the word 'detection' is used with two meanings in the paper (the other being the localization of the diatom in an image). Perhaps the term 'out-of-distribution recognition' could be used instead.6) It seems that the OOD detection experiment does not include non-diatom particles. As the original images contained background particles (sediment, clay, diatom fragments, etc.), it would have been beneficial to include them as OOD samples. This would provide a more realistic experiment setup.7) It is unclear how the

baseline method is applied in the OOD experiment. Is it thresholding of softmax probabilities? Training a binary classifier with the OOD samples in the training set?8) I don't think AUROC and AUPR are very good evaluation metrics as the numbers are very hard to interpret (what does an AUROC of 0.8388 actually mean in practice?). Typically, we want to find the best decision threshold and are interested in classification accuracy with that threshold.9) The abbreviation SSL is used with two different meanings: semi-supervised learning (e.g., page 14) and self-supervised learning (e.g., page 16). The same applies to the description of what the second experiment is about. While self-supervised learning can be utilized in semi-supervised learning, they are definitely not the same thing.10) What are micro- and macro-average in Table 4?"

Level of Interest

Please indicate how interesting you found the manuscript: Choose an item.

Quality of Written English

Please indicate the quality of language in the manuscript: Choose an item.

Declaration of Competing Interests

Please complete a declaration of competing interests, considering the following questions:

- Have you in the past five years received reimbursements, fees, funding, or salary from an organisation that may in any way gain or lose financially from the publication of this manuscript, either now or in the future?
- Do you hold any stocks or shares in an organisation that may in any way gain or lose financially from the publication of this manuscript, either now or in the future?
- Do you hold or are you currently applying for any patents relating to the content of the manuscript?
- Have you received reimbursements, fees, funding, or salary from an organization that holds or has applied for patents relating to the content of the manuscript?
- Do you have any other financial competing interests?
- Do you have any non-financial competing interests in relation to this paper?

If you can answer no to all of the above, write 'I declare that I have no competing interests' below. If your reply is yes to any, please give details below.

I declare that I have no competing interests

I agree to the open peer review policy of the journal. I understand that my name will be included on my report to the authors and, if the manuscript is accepted for publication, my named report including any attachments I upload will be posted on the website along with the authors' responses. I agree for my

report to be made available under an Open Access Creative Commons CC-BY license (http://creativecommons.org/licenses/by/4.0/). I understand that any comments which I do not wish to be included in my named report can be included as confidential comments to the editors, which will not be published.

Choose an item.

To further support our reviewers, we have joined with Publons, where you can gain additional credit to further highlight your hard work (see: https://publons.com/journal/530/gigascience). On publication of this paper, your review will be automatically added to Publons, you can then choose whether or not to claim your Publons credit. I understand this statement.

Yes Choose an item.
